# Direct 3D Mass Spectrometry Imaging Analysis of Environmental Microorganisms

**DOI:** 10.3390/molecules30061317

**Published:** 2025-03-14

**Authors:** Justyna Szulc, Tomasz Grzyb, Joanna Nizioł, Sumi Krupa, Wiktoria Szuberla, Tomasz Ruman

**Affiliations:** 1 Department of Environmental Biotechnology, Faculty of Biotechnology and Food Sciences, Lodz University of Technology, 90-530 Lodz, Poland; justyna.szulc@p.lodz.pl; 2 Department of Inorganic and Analytical Chemistry, Faculty of Chemistry, Rzeszow University of Technology, 35-959 Rzeszow, Poland; jniziol@prz.edu.pl (J.N.); d605@stud.prz.edu.pl (S.K.); 163834@stud.prz.edu.pl (W.S.); tomruman@prz.edu.pl (T.R.)

**Keywords:** 2D/3D microbiological culture imaging, mass spectrometry imaging, microbial interactions, phytopathogens, bioactive compounds, metabolomic analysis

## Abstract

Assessing the spatial distribution of microorganisms’ metabolites in growth medium remains a challenge. Here, we present the first use of the newly developed LARAPPI/CI-MSI 3D (laser ablation remote atmospheric pressure photoionization/chemical ionization mass spectrometry imaging) method for direct three-dimensional (3D) mass spectrometry imaging of bacterial and fungal metabolites in solid culture media. Two-dimensional (2D) MSI was also performed, and it indicated the presence of metabolites belonging to, and including, amino acids and their derivatives, dipeptides, organic acids, fatty acids, sugars and sugar derivatives, benzene derivatives, and indoles. Distribution at a selected depth within the culture medium with the estimation of concentration across all dimensions of 16 metabolites was visualized using LARAPPI/CI-MSI 3D. The imaging results were correlated with the results of ultra-high-performance liquid chromatography–ultra-high-resolution mass spectrometry (UHPLC–UHRMS). A total of 351–393 chemical compounds, depending on the tested microorganism, were identified, while 242–262 were recognized in the HMDB database in MetaboAnalyst (v 6.0). The LARAPPI/CI-MSI 3D method enables the rapid screening of the biotechnological potential of environmental strains, facilitating the discovery of industrially valuable biomolecules.

## 1. Introduction

Microorganisms are a valuable source of primary and secondary metabolites with industrial potential [1,2]. Primary (central) metabolites include amino acids, nucleotides, and fermentation end products such as ethanol and organic acids. They are produced by energy metabolism and are responsible for the growth, development, and reproduction of microorganisms [2]. Secondary metabolites are compounds produced by the modification of primary metabolite synthases, usually at the end of stationary growth. They include pigments, mycotoxins, antibiotics, and others. Numerous secondary metabolites play decisive roles in ecological functions, in particular, mediating antagonistic interactions [1].

Microbial metabolites are key to the production of many food products, dietary supplements, vitamins, amino acids, organic acids, agriculturally important metabolites, enzymes, flavoring agents, coloring agents, and pharmaceutical products [3,4,5]. Examples of industrially used metabolites include amino acids from *Corynebacterium*, *Brevibacterium* and *Escherichia coli* bacteria; vitamins from *Propionibacterium* and *Pseudomonas*; organic acids produced by *Aspergillus*, *Lactobacillus*, *Rhizopus* molds, and enzymes from *Aspergillus* and *Bacillus* [4,6,7,8,9]. Secondary metabolites are widely used in health care as anabolic agents, anesthetics, anti-inflammatory agents, antithrombotic agents, hemolytic, vasodilators, hypocholesterolemic agents, antimicrobials, antiparasitics, anticancer agents, enzyme inhibitors, and immunosuppressive drugs [10,11,12]. Some secondary metabolites show plant growth stimulating effects or herbicide and insecticide activity [13]. Anti-cancer effects have also been demonstrated for adriamycin, bleomycin, daunomycin, and mithramycin, among others [14,15].

The advancement of biotechnological production is driven by its environmental benefits, including a lower pollution burden compared to conventional chemical methods, as well as societal pressure for sustainable resource exploration [4]. Various strategies have been identified for effective overproduction of microbial metabolites through genetic and physiological manipulation [16]. These include the overexpression of genes responsible for the synthesis of metabolites and related coenzymes, the knockdown of genes involved in degradation, and continuous extraction of metabolites from cultures [17]. However, more effective tools are needed for rapid assessment of the metabolic abilities of environmental microorganisms.

Recently, along with the development of other omics methods (genomics and transcriptomics), there has been an expansion of metabolomics, including MSI (mass spectrometry imaging) methods in microbiological studies [18,19]. The most commonly used method is matrix-assisted laser desorption/ionization mass spectrometry imaging (MALDI MSI). This method has been used to identify microorganisms based on the ribosomal protein profile and reference databases [20,21,22], as well as for the identification and imaging of metabolites of microbiological origin (including mycotoxins) on technical and historical materials [19,23,24].

Since MS imaging of microorganism structures and their growth media is currently performed almost exclusively in 2D, a key challenge remains the development of 3D analysis techniques [25]. So far, 3D imaging has been most often performed using specialized software that stacks multiple 2D results. Recently, Shein et al. [26] proposed a moisture-assisted cryo-section (MACS) modification of MALDI-MSI. However, this method requires sectioning colonies and maintaining appropriate humidity conditions.

The laser ablation remote atmospheric pressure photoionization/chemical ionization (LARAPPI/CI) platform coupled to an ultra-high-resolution quadrupole-time-of-flight (QToF) mass spectrometer (LARAPPI/CI MSI system) was described in a recent publication by Ruman and co-workers [25]. The setup contains an airtight chamber with flowing nitrogen gas (10 L/min). The sample is placed on a sample stage with a Peltier cooling plate for sample freezing. The sample stage is mounted on a motorized XYZ-configuration stage. The pulses from the OPO laser (2.93 µm) passes through a diffractive optical element, forming a square-shaped top-hat beam that is focused onto the sample surface by a 50 mm focal length aspherical ZnSe lens. The system contains also the camera with a lens and distance sensor, the latter for the 3D profiling of the sample shape and dimensions. During MSI, the laser ablation plumes are taken with the gas and transported to the ion source (Bruker VIP HESI in the APCI configuration) of the Bruker Impact II mass spectrometer (Bruker Daltonics, Bremen, Germany). An HPLC pump provides the solvent mixture (1% toluene in methanol; 200 μL/min) to the APCI needle. This system is capable of performing 2D and also direct 3D MSI.

The LARAPPI/CI MSI technique offers several noteworthy advantages over more established methods such as desorption electrospray ionization (DESI), matrix-assisted laser desorption/ionization (MALDI), and surface-assisted laser desorption/ionization (SALDI). From a practical standpoint, LARAPPI/CI can be performed under ambient conditions—akin to DESI—and does not necessarily require sample slicing into ultra-thin sections or the use of exogenous matrix layers. This feature helps preserve the native configuration of many biological specimens, including plant or animal tissues, especially those that contain regions of markedly different structural integrity (e.g., hydrated gel-like areas). Compared to MALDI, which commonly uses vacuum conditions and an organic or nanoparticle-based matrix, LARAPPI/CI does not require a vacuum system for desorption, minimizing potential sample deformation due to dehydration. This method was recently used by Ruman et al. [25,27] for the first 2D and 3D mass spectrometry imaging (MSI) of metabolites in human and plant tissues.

The aim of this study was to evaluate the usefulness of the newly developed LARAPPI/CI-MSI 3D (laser ablation remote atmospheric pressure photoionization/chemical ionization mass spectrometry imaging) method for direct 3D mass spectrometry imaging of bacterial and fungal metabolites in solid culture media. Two-dimensional (2D) MSI was also performed to study the localization of fungal and bacterial metabolites, including amino acids and their derivatives, dipeptides, organic acids, fatty acids, sugars and sugar derivatives, benzene derivatives, and indoles. Here, we present the first direct 3D MSI of solid bacterial and fungal cultures. We show that direct 3D mass spectrometry imaging analysis can also be used for metabolome analysis of microbial cultures, creating new opportunities for the metabolic profiling of industrially important strains.

## 2. Results and Discussion

### 2.1. 2D Mass Spectrometry Imaging of Microbial Cultures

The LARAPPI/CI-MSI 2D was used to visualize numerous metabolites of bacterial and fungal origin in agar cultures, including six dipeptides and one dipeptide derivative (PyroGlu-Ala) (Table 1). Some of the metabolites (Pro-Leu; PyroGlu-Ala; Pro-Asn) occurred with different intensities, depending on whether they originated from bacterial or fungal cultures. Others (Pro-Pro; Pro-Val; Leu-Pro) were associated with a bacterial colony (*B. cereus*). Asp-Leu was associated with a fungal colony (*F. graminarum*).

LARAPPI/CI-MSI 2D facilitated the imaging of a wide range of important amino acids from the point of view of biochemistry and medical and industrial biotechnology (Table 2). L-glutamine has many practical applications, primarily in the food and pharmaceutical industries. Glutamine has been shown in laboratory simulations to have numerous benefits in the treatment of critical illnesses, cancer, and cardiac injury. The mechanism of action of glutamine includes improving immune cell function, strengthening the intestinal barrier, increasing stress tolerance, reducing the expression of proinflammatory cytokines, and lowering mortality [28]. Glutamine is an amino acid used as a dietary supplement to support the immune system, increase glycogen synthesis, exert anticatabolic effects, and enhance fluid and electrolyte uptake [29]. L-glutamine is produced using bacteria from the genus *Bacillus* and *Paenibacillus*. Therefore, the presented method can be used to assess the industrial potential of these bacteria for glutamine production. Similarly, L-arginine is currently used in pharmacology for the treatment of liver dysfunction, metabolic alkalosis, ammonia poisoning, asthenic disorders and malnutrition, and growth hormone deficiency. It is also used as a supplement for athletes. It can potentially be used in the treatment of various types of hypertension, heart disease, atherosclerosis, hypercholesterolemia, glaucoma, renal failure, diabetes, and other diseases [30].

One of the main metabolites of *B. coagulans* R11 is 4-acetamidobutanoic acid, which shows antioxidant and antimicrobial activity [31]. L-glutamic acid can be produced with high yield by environmental isolates of *Bacillus* sp. [32]. L-glutamic acid in the form of monosodium salt is commonly used as a flavor-enhancing compound in food [33]. Similarly, *N*-acetylglycine has been found at low concentrations in various types of food products. It is not genotoxic in in vivo or in vitro assays [34]. Diaminopimelic acid is a constituent of the bacterial and fungal cell wall structure and is found in higher plants [35]. L-proline is an osmoprotectant that takes part in the response of bacteria and plants to osmotic stress. It also has many other functions, including as an antioxidant that captures reactive oxygen species. It increases the stability of proteins and membranes during freezing, dehydration, or heating. Additionally, it can increase the solubility of some proteins and inhibit protein aggregation [36]. Aspartic acid has potential antibacterial and antifungal effects [37]. Citrulline is a precursor of arginine and plays an important role in the metabolism and regulation of nitric oxide. Citrulline supplements may help to control disorders of NO metabolism and improve cardiovascular function [38]. Tryptophan is an essential amino acid in mammals. It is involved in the proper functioning of neurons, the immune system, and the intestines. Tryptophan supplementation may be beneficial for diseases such as autism, cardiovascular disease, chronic kidney disease, depression, inflammatory bowel disease, multiple sclerosis, and bacterial infections, among others [39,40]. Creatine plays a key role in cellular metabolism, especially in states of metabolic stress. Its main practical application is as an ergogenic agent for exercise, training, and sports, improving cellular metabolism and reducing the severity of injuries and/or disease states [41]. In turn, 2-aminoadipic acid is a key intermediary in the biosynthesis of lysine and penicillin in microorganisms producing β-lactams and 3-lactams. Additionally, it plays a role in lysine catabolism in mammals [42]. The remaining amino acids and their derivatives imaged by this method are listed in Supplementary Materials Table S1.

The LARAPPI/CI-MSI 2D technique also revealed a wide range of organic acids produced by the studied microorganisms (Table 3). Organic acids exert antimicrobial activity by lowering the environmental pH and lowering the internal pH of the microbial cells through ionizing undissociated acid molecules. Additionally, they disrupt substrate transport by changing the permeability of the cell membrane or reducing the proton driving force [43]. Pyrrolidone carboxylic acid is a valuable compound used in many cosmetics due to its moisturizing properties and ability to retain moisture (Villeneuve et al., 2003) [44]. It is also known for its antimicrobial properties, including against bacteria of the genera *B. subtilis, Escherichia, Enterobacter*, *Klebsiella*, and *Pseudomonas* [45]. Malic acid has been shown to have antibacterial activity against food pathogens (*Listeria monocytogenes*, *Salmonella enterica*, and *Escherichia coli*) [46]. It can also stimulate the production of *Fusarium* antagonists in soil [47]. Similarly, citric acid has proven activity against foodborne pathogens including *Shigella* sp., *Listeria*, *Escherichia*, and *Salmonella* [43,48].

Pantothenic acid has many applications in the food, cosmetic, and pharmaceutical industries. Its biotechnological production methods are well established [49]. Propionic acids are weak organic acids, which are commonly used as preservatives in various food products. The antimicrobial activity of succinic acid has been applied for the preservation of poultry and beef meat [50]. Several studies have reported that lactic acid of microbiological origin has antimicrobial activity against bacteria and molds, both in vitro and in foods [51]. Unver [52] showed that fumaric acid has a potent inhibitory effect against pathogenic and opportunistic microorganisms, including bacteria belonging to the genus *Escherichia*, *Enterobacter*, *Klebsiella*, and *Pseudomonas*, as well as *Candida* yeast. It also has potential analgesic, anti-inflammatory, antioxidant, antipsoriatic, chemopreventive, immunomodulatory, and neuroprotective properties. Hydroxypropanedioic acid detected in the present study in the *B. cereus* colony is a primary metabolite directly involved in the growth, development, and reproduction of living organisms. 3-hydroxypropionic acid is an industrially valuable compound used in the chemical synthesis of solvents, cleaning agents, adhesives, paints and coatings, plastics, and fibers. Genetic engineering has been used to enable its microbial production, including from bacteria of the genus *Bacillus* [53].

Dihydroxyfumaric acid is used for the biosynthesis of sugars, uronic acids, and vitamin C. Its industrial applications include its use as a disinfectant in contact lenses and as a color-destabilizing agent in cleaning products. It can also be used to treat municipal wastewater or wastewater from the paper or food industries [54]. 2-aminobenzoic acid from *Bacillus* has been identified as compound involved in microbial induced systemic resistance, demonstrating activity against soft-rot disease in tobacco [55]. Sebacic acid (decanedioic acid) has antimicrobial and antifungal activity and has been reported previously in *Bacillus* species. 2-furoic acid demonstrates strong nematicidal activity [56]. Xanthurenic acid is produced by bacteria as part of tryptophan catabolism. It is associated with the endogenous cell death factor, which accelerates the cell-aging process [57]. Depending on the microbe, the function of kynurenic acid is unclear. This compound is considered to play an essential role in the survival of microorganisms, including amino acid synthesis and nitrogen assimilation [58].

Notably, azelaic acid was one of the fatty acids and derivatives detected by LARAPPI/CI-MSI 2D (Table 4). Azelaic acid has anti-inflammatory, antibacterial, antioxidant, anti-comedogenic, and anti-cancer effects. It has been approved by the US Food and Drug Administration as a therapeutic agent for the treatment of acne and rosacea [59]. It is a natural inducer of the plant defense system, capable of stimulating innate resistance in plants and reducing the damage caused by diseases, without the negative effects of fungicides [60]. Azelaic acid is also a valuable bio-based monomer for the synthesis of biodegradable and sustainable polymers, plasticizers, and lubricants [61]. Linoleic and linolenic acids also have broad antibacterial activity [62]. Pentadecanoic acid has anti-biofilm properties, including against *Klebsiella pneumoniae* bacteria and *Candida albicans* fungi [63]. Bacteria of the *Bacillus* genus are known to produce valeric acid [64]. Its effectiveness against phytopathogens of the *Fusarium* genus as well as Gram-negative and Gram-positive bacteria has also been demonstrated [65].

This is the first study to report finding elaidic acid on the edge of the *Bacillus* growth area. The probable explanation is that this acid was synthesized due to the presence of high concentrations of toxic substances from *F. graminarum* [66]. It seems that the *s*ecretion of elaidic acid at the edge of the culture modified the membrane fluidity as a defense mechanism. Elaidic acid is known to have an antimalarial effect [67].

Other biotechnologically important sugars and their derivatives were also imaged using LARAPPI/CI-MSI 2D (Table 5).

Corona and Munday [68] found ribitol in the mycelium of molds, but, as in the present study, they did not observe any diffusion into the culture medium. Similarly, in our work, the intensity of signals indicates that this compound was produced in small amounts and was concentrated in the colonies rather than in the medium. Onose et al. [69] reported that bacteria of the genus *Bacillus* (*Bacillus amyloliquefaciens* and *Bacillus subtilis*) produce 2-amino-2-deoxy-D-mannitol, which is a precursor of 1-deoxynojirimycin and has therapeutic applications in the treatment of HIV, Gaucher’s disease, and diabetes. Interestingly, the level of sorbitol in the culture medium increased with the production of this compound. Sorbitol was one of the metabolites of *F. graminarum* co-cultured with *P. amylolyticus*. It is a natural polyol used as a sweetener, moisturizing agent, and softener. It can be produced chemically or biotechnologically using microorganisms, primarily *Zymomonas mobilis* [70]. Observing the interactions of the metabolites of both groups of microorganisms may be useful for optimizing the production of therapeutic metabolites.

4-amino-4-deoxy-L-arabinose is an important metabolite for the modification of lipid A, which is a component of the lipopolysaccharides of Gram-negative bacteria. It participates in the acquisition of resistance to polymyxin antibiotics [71]. Batovska et al. [72] proposed erythronic acid and other individual metabolites as biomarkers for fungal resistance based on the correlations between GC/MS data and the estimated resistance of leaves towards the etiological agents of powdery mildew and downy mildew. 2-deoxyriobse 5-phosphate is used as a building block for antisense and antiviral drugs, reagents, and reagents for PCR [73]. *N*-acetylmannosamine is a plant primary metabolite precursor with bioprotective features [74]. *N*-acetylmannosamine induces gratuitous β-*N*-acetylhexosaminidase in *C. albicans* and *Mucor fragilis*, which performs an important vegetative function in heterotrophic microorganisms [75]. Trehalose is widely present in bacteria, fungi, plants, and invertebrates. In fungi, trehalose constitutes a source of reserve carbon and agents of adaptation to stress conditions, such as dehydration, oxidative, and heat/cold treatment. Trehalose is capable of stabilizing and protecting membranes and proteins, making it suitable for use as a protective agent during the freeze-drying of microorganisms [76].

The LARAPPI/CI-MSI 3D method also enabled the acquisition of ion images for other bacterial and fungal metabolites, including alcohols, indoles, amines, aldehydes, purines, pyrimidines, benzene derivatives, organic phosphates, carnitines, and sulfur compounds (Table S1).

### 2.2. 3D Mass Spectrometry Imaging of Microbial Cultures

This is the first study to use the LARAPPI/CI-MSI 3D method to generate ion images of bacterial and fungal metabolites in solid culture media (Table 6). The method facilitated the assessment of chemically diverse metabolites, classified as amino acids and amino acid derivatives, dipeptides, organic acids, fatty acids, sugars and sugar derivatives, benzene derivatives, and indoles. A novelty of the method is that it enables the visualization of chemical compound distribution at a selected depth within the culture agar while also allowing for the estimation of compound concentration across all dimensions. The 3D ion images for L-glutamine, L-glutamic acid, L-arginine, and *N*-acetylglycine match those obtained in the 2D analysis. This indicates the presence of these compounds in both types of colonies (bacterial and fungal), although their distributions are different. The concentration of L-glutamine was higher in the center of the fungal colony and in the outer part of the bacterial colony. The concentration of glutamic acid was higher in *Paenibacillus* than in *Fusarium*. L-arginine was detected only in bacteria. Similar to indole-3-carboxylic acid, 3D analysis showed a better diffusion of diaminopimelic acid into the substrate than 2D analysis and an almost uniform distribution in the culture medium, making it difficult to assign this metabolite (Table 6).

According to the results of 3D analysis, malic acid is characteristic for molds, and pyrrolidonecarboxylic acid dominates in the bacterial colony. Pro-Leu cannot be considered a microorganism-specific dipeptide because it occurred in almost the entire volume of the tested area. In contrast, azelaic acid is closely related to *Paenibacillus,* and the obtained ion image suggests that it is not released into the substrate. The applied method also proved effective for imaging sugars and sugar derivatives from molds, revealing the spatial distribution for ribitol, sorbitol, and deoxyguanosine. As can be seen more clearly from the 3D ion image for *N*-methylbenzamide, this metabolite is primarily associated with the bacterial colony but probably also occurs in the culture medium itself (Table 6). Additionally, 3D ion images were generated for D-α-aminobutyric acid and *N*-acetyl-L-alanine (Table S2). Moreover, MS spectra of the top ablation level of 3D MSI are provided in Supplementary Materials Figure S1.

It is worth emphasizing that the advantage of LARAPPI/CI in the context of comparing MSI methods is its suitability for imaging microbial colonies cultured on agar-based media. Owing to the mid-infrared laser’s strong coupling with water, LARAPPI/CI enables efficient desorption of microorganisms and surrounding matrix without requiring the vacuum conditions typical of SIMS (secondary-ion mass spectrometry) or MALDI. The sample can remain in a near-native state—particularly important for microbial colonies whose morphology and metabolite gradients can be distorted by drying, slicing, or excessive handling.

### 2.3. Pathway Analysis

**Figure 1:**
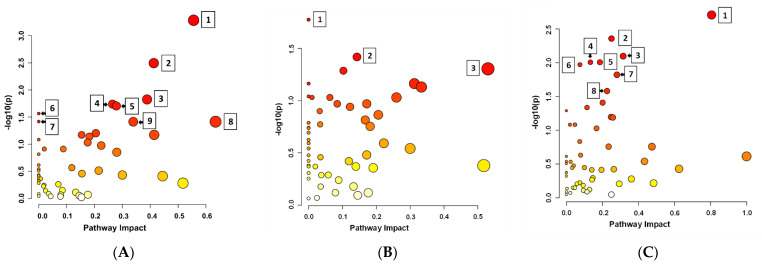
Pathway impact analysis of metabolites for the tested microorganisms. The bubble plot shows metabolic pathways impacted based on pathway impact scores (*X*-axis) and −log10(*p*) values (*Y*-axis). Larger and more intense red bubbles represent pathways with higher impact and statistical significance. Pathways numbered in the plot have the greatest influence and significance in distinguishing between the groups. (**A**) *Paenibacillus amylolyticus*; 1—D-amino acid metabolism; 2—arginine biosynthesis; 3—arginine and proline metabolism; 4—glycine, serine, and threonine metabolism; 5—purine metabolism; 6—cyanoamino acid metabolism; 7—tyrosine metabolism; 8—alanine, aspartate, and glutamate metabolism; 9—pyrimidine metabolism; (**B**) *Bacillus cereus*, 1—tyrosine metabolism; 2—taurine and hypotaurine metabolism; 3—alanine, aspartate, and glutamate metabolism; (**C**) *Fusarium graminarum*; 1—alanine, aspartate, and glutamate metabolism; 2—arginine biosynthesis; 3—purine metabolism; 4—butanoate metabolism; 5—lysine biosynthesis; 6—glyoxylate and dicarboxylate metabolism; 7—pyruvate metabolism; 8—citrate cycle (TCA cycle).

After LC-MS positive and negative mode analysis, 382 chemical compounds were identified for *P. amylolyticus*, of which 262 metabolites were included in the analysis using the *Bacillus subtilis* subsp. *subtilis 168* (KEGG) library. In turn, 351 chemical compounds were detected for *B. cereus*, of which 242 metabolites were included in the analysis using the same library. For *F. graminarum*, 393 chemical compounds were identified, of which 244 metabolites were included in the analysis using the *Aspergillus clavatus* library (KEGG). Figure 1 shows a pathway impact analysis of metabolites for the tested microorganisms. The most statistically significant pathways for both tested bacteria strains were tyrosine, alanine, aspartate, and glutamate metabolism. For *B. cereus*, only the taurine and hypotaurine metabolism pathway was statistically significant. More significant metabolic pathways were detected for *P. amylolyticus*, including D-amino acid metabolism; arginine biosynthesis; arginine and proline metabolism; glycine, serine and threonine metabolism; purine metabolism; cyanoamino acid metabolism. For *F. graminarum*, the most important pathways were as follows: alanine, aspartate, and glutamate metabolism; arginine biosynthesis; purine metabolism; butanoate metabolism; lysine biosynthesis; glyoxylate and dicarboxylate metabolism; pyruvate metabolism; the citrate cycle (TCA cycle).

**Figure 2:**
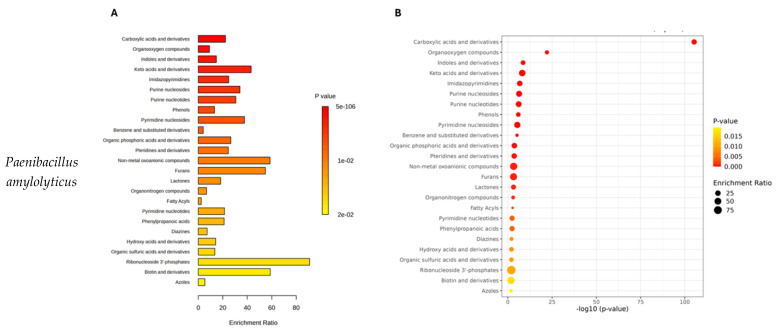

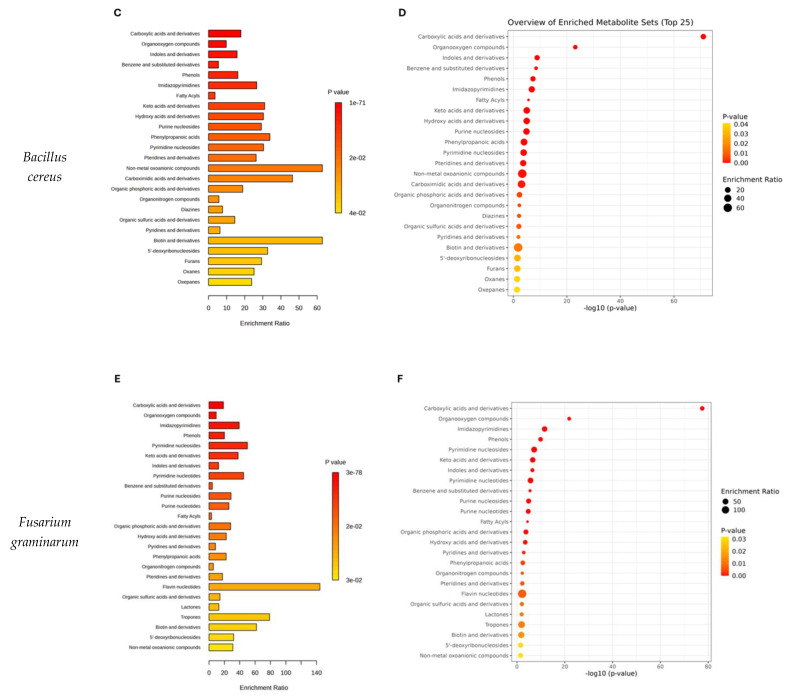
Pathway enrichment analysis results for the tested microorganisms based on chemical compound classes. (**A,C,E**) Bar plots of enriched chemical classes showing the enrichment ratio for each class. (**B,D,F**) Bubble plots illustrating the pathway impact with the corresponding −log10(*p*-value). (**A**,**B**) data for *Paenibacillus amylolyticus,* (**C**,**D**) data for *Bacillus cereus*, (**E**,**F**) data for *Fusarium graminarum*. Larger bubbles represent higher enrichment ratios. The color scale indicates statistical significance (*p*-value), with red indicating higher significance.

The identified metabolic pathways, including the key metabolites and their statistical significance, are presented in Tables S3–S5. The results of pathway enrichment analysis results are presented in Figure 2.

In total, 34 metabolite sets were identified for *P. amylolyticus*, and 38 sets were identified for *B. cereus* (Supplementary Materials Tables S6 and S7). For *F. graminarum,* 37 metabolite sets were found (Table S8). The bacteria metabolite profile was dominated by carboxylic acids and derivatives (39.9% of detected compounds for *P. amylolyticus* and 32.2% for *B. cereus*), organooxygen compounds (13.8% and 14.8%), benzene and substituted derivatives (5.9% and 8%), fatty acyls (5.5% and 8%), and indoles and derivatives (4.0% and 4.2%). The remaining metabolite sets accounted for less than 2.8% of hits in the *P. amylolyticus* profile and less than 3.4.% for *B. cereus* (Figure 2A,B, Tables S6 and S7). Similarly, the dominant metabolites detected for the mold *F. graminarum* were carboxylic acids and derivatives (33.8%), organooxygen compounds (14.2%), fatty acyls (7.1%), benzene and substituted derivatives (6.2%), and indoles and derivatives (3.3%). Large proportions of phenols (4.2%) and imidazopyrimidines (3.8%) were also detected, while other metabolites accounted for less than 2.1% (Figure 2C, Table S8). The most discriminating compounds between analyzed microorganism sets were visualized using volcano plots (Figures S2 and S3). Furthermore, the differences in the abundance of these compounds are illustrated by box plots (Figures S4 and S5).

## 3. Materials and Methods

### 3.1. Microorganisms

The study used two strains of genetically identified environmental bacteria: *Paenibacillus amylolyticus* (NCBI genome assembly number ASM4051371v1) and *Bacillus cereus* (NCBI genome assembly number ASM4309929v1). The strains were isolated from soil (corn plantation located in Central Poland, 2023 year). The strains were deposited in the collection of pure cultures of the Department of Environmental Biotechnology, Lodz University of Technology (Lodz, Poland). The tested mold strain was *Fusarium graminarum* IOR 1540 (one of the most common fungal pathogens of maize). The strains were sourced from the Institute of Plant Protection—National Research Institute in Poznan (Poznan, Poland).

### 3.2. Microbial Cultures

**Figure 3:**
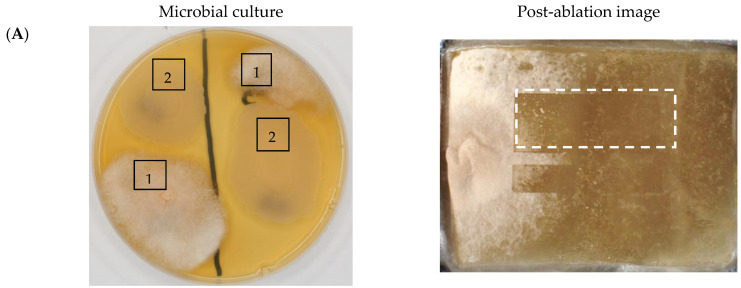

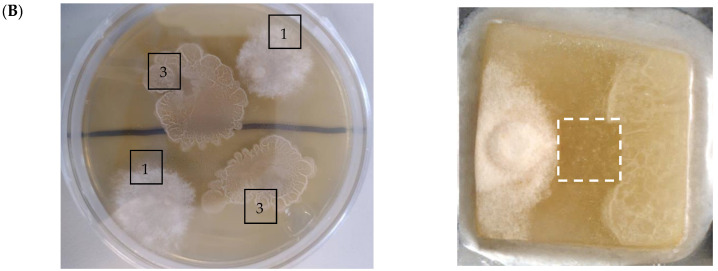
Tested agar medium samples used in MSI experiments: (**A**) *Fusarium graminarum* (1) and *Bacillus cereus* (2); (**B**) *Fusarium graminarum* (1) and *Paenibacillus amylolyticus* (3). Dashed line areas represent imaged regions.

The bacteria were cultured together with the mold strain (in pairs). Cultures were prepared pointwise with a distance of 5 cm between the bacteria and mold on petri dishes containing TSA agar medium (Tryptic Soy Agar, Merck-Millipore, Poznan, Poland). Samples were prepared in four replicates. The microorganisms were incubated at 30 ± 2 °C for 3 days. Portions of the agar medium with microorganisms measuring 20–30 × 20–30 mm in size (shown in Figure 3) were cut using stainless steel blades and transferred to stainless steel plates (45 × 35 × 0.8 mm) for use in MSI experiments.

### 3.3. Mass Spectrometry Imaging (MSI) Experiments

#### 3.3.1. LARAPPI/CI MSI System

The laser ablation remote atmospheric pressure photoionization/chemical ionization (LARAPPI/CI) platform coupled to an ultra-high-resolution quadrupole-time-of-flight (QToF) mass spectrometer (LARAPPI/CI MSI system) first described in a recent publication [25] is based on an airtight chamber pressurized with nitrogen gas to produce a nitrogen stream of 10 L/min. The sample (Figure 3) is placed on a 50 × 50 mm sample stage, with a Peltier cooling plate that sustains the sample at −18 °C. The temperature-controlled sample stage is mounted on a motorized high-speed XY stage. The pulsed beam from the OPO laser (2.93 µm, 7 ns, 20 Hz, 3.5 mJ/pulse) enters the sample chamber through sapphire window. Then, the beam is expanded 3.75× and is redirected toward the sample stage by a gold mirror. The beam then goes through a diffractive optical element forming square-shape top-hat beam. It is then focused onto the sample surface by a 50 mm focal length aspherical ZnSe lens (ThorLabs, Mölndal, Sweden). The optical assembly and also the camera with the lens and distance sensor are mounted on aluminum rails and are in a fixed configuration; the only moving parts are XYZ stages. During imaging, the laser focal point remains fixed in space, whereas the sample is moved. A specially designed gas funnel is also a focusing assembly and is connected to a 6/4 mm (O.D/I.D.) PTFE tube. The overpressure in the chamber drives a 10 L/min nitrogen gas flow through the tube. The laser ablation plumes are entrained into the gas and transported to the modified ion source (Bruker VIP HESI in the APCI configuration) of the Bruker Impact II mass spectrometer (Bruker Daltonik GmbH, Bremen, Germany). The ion source also had a VUV source (Hamamatsu L12542) (Hamamatsu Photonics K.K., Iwata City, Japan) mounted axially to the MS sampling cone inside the ion source. A HPLC pump (Agilent G1312A) (Agilent Technologies, Santa Clara, CA, USA) provides a steady flow of a solvent mixture (1% toluene in methanol; 200 μL/min) to the APCI needle [25]. The settings of the ion source were as follows: APCI nebulizer, end-plate offset 600 V, capillary 1000 V, corona 6000 nA, nebulizer 3.5 bar, dry gas 0.2 L/min, dry temperature 250 °C, probe gas temperature 350 °C, probe gas 4 L/min, exhaust turned on. MS1 experiments were performed with the following settings: scan range *m*/*z* 47–1300.

#### 3.3.2. Direct Three-Dimensional Mass Spectrometry Imaging (MSI 3D)

The spatial resolution for 3D MSI experiment was 140 µm with applied oversampling. Each pixel/voxel in 3D MSI experiments was exposed to the laser for 0.5 s, at a laser pulse repetition rate of 20 Hz. The delays between pixels were 1200 ms. Between pixels, the sample stage moved at a speed of 50 mm/s. The time delay between lines was 3 s. Each 3D experiment was carried out in an inverted pyramid ablation scheme [25].

The 3D MSI experiment (Figure 3B, Table 6) performed on sample containing *Paenibacillus amylolyticus* and *Fusarium graminarum* had a top ablation level of 35 × 35 (X × Y) voxel arrangement, second (lower) level—34 × 34, with ablation levels at 3.25, 3.00 mm for top level and lower levels, respectively. The starting object was of ca. 3.25 mm thickness and 19.2 × 18.6 mm (X × Y) size, while the analyzed area was of 4.8 × 4.8 mm (X × Y) size. The objects were cut with a blade and placed on a stainless-steel plate and then on an ablation table inside the chamber and frozen.

#### 3.3.3. Two-Dimensional Mass Spectrometry Imaging (MSI 2D)

The spatial resolution for 2D MSI experiment was 240 µm without oversampling. Each pixel/voxel in 2D MSI experiments was exposed to the laser for 1 s at a laser pulse repetition rate of 20 Hz. The delays between pixels were 1200 ms. Between pixels, the sample stage moved at a speed of 50 mm/s. The time delay between lines was 5 s [25]. The 2D MSI experiment (Figure 3A) performed on sample containing *Paenibacillus amylolyticus* and *Fusarium graminarum* had a top ablation level of 77 × 31 (X × Y) voxel arrangement, with the ablation level at 4.50 mm. The starting object was of ca. 4.5 mm thickness and 34 × 28 mm size, and the analyzed region was 18.2 × 7.2 mm (X × Y) size. The objects were cut with a blade and placed on a stainless-steel plate and then on an ablation table inside the chamber and frozen.

### 3.4. Ultra-High-Performance Liquid Chromatography–Ultra-High-Resolution Mass Spectrometry (UHPLC–UHRMS)

#### 3.4.1. LC–MS Sample Preparation

Metabolomic profiling was performed on medium samples from both regions of each Petri dish with microorganism growth. Approximately 200 mg of the sample was weighed and cut into small pieces. To the sample pieces, 150 μL of distilled water and 900 μL of acetone (Sigma-Aldrich, Poznan, Poland LC–MS grade) were added. At the same time, 3 stainless beads were added to this suspension, and the samples were homogenized 3 times for one minute with BeadBug 6 (Benchmark Scientific, Sayreville, NJ, USA) at 4000 rpm. Then, the samples were incubated at temp. 4 °C overnight. The following day, the samples were centrifuged (mySPIN™ 12 Mini Centrifuge, Thermo Fisher Scientific, Waltham, MA, USA; temp. 4°C, 14,000× *g*, 5 min). The supernatant of each sample was transferred, and the samples were left in a speed vac-type apparatus (2 × 10^−3^ mbar vacuum) overnight. The next day, the dried pellet was dissolved in methanol (800 μL). To facilitate dissolution, the samples were sonicated and then centrifuged for 5 min at 14,000× *g*, and the resulting supernatants were transferred to a standard HPLC vials.

#### 3.4.2. LC–MS Metabolomic Analysis

Mass spectrometry–liquid chromatography analyses were performed on a Bruker Elute UHPLC system operated by Hystar 3.3 software and an ultra-high-resolution (60,000+) mass spectrometer Bruker Impact II (Bruker Daltonik GmbH, Bremen, Germany) ESI QTOF-MS equipped with Data Analysis 4.2 (Bruker Daltonik GmbH, Bremen, Germany), TASQ (2022b), and Metaboscape (2022b). The ion source used was a Bruker VIP-HESI with optimized flows and temperatures. The column used for AutoMSMS measurements was Waters Acquity UPLC BEH C18 1.7 µm particles of 2.1 × 50 mm dimensions (Waters Poland, Gdansk, Poland). For AutoMSMS measurements flows and percentages were 0 and 0.56 min 99% A, 4.72 min—1% A, 5.56 min—1% A, and 5.60, 6.34, and 9.45 min—99% A, all flows at 450 μLmin^−1^ (Ossoliński et al., 2024; Szulc et al., 2024; Nizioł et al., 2024) [77,78,79]. The column was held at 40 °C.

The column exit was connected to the VIP HESI ion source. Internal calibration on 10 mM sodium formate (water–isopropanol 1:1 *v*/*v*) ions was performed automatically in Metaboscape with the use of a syringe pump at an infusion flow rate of 0.12 mL h^−1^, using a high-precision calibration (HPC) mode. The autoMSMS method was used with an *m*/*z* range 50–1500; CID (Collision-Induced Dissociation) was used with the following settings: absolute area threshold: 5000 counts; active exclusion 2 spectra; release after 0.3 min, isolation mass: for *m*/*z* = 100, the width was 4; for 300, the width was 5; for 500, the width was 6; for 1000, the width was 8; collision energy value was 30 eV.

The untargeted annotations were performed in Metaboscape (ver. 2022b) with a criterion of mass deviation (∆*m*/*z*) under 2 ppm and mSigma value (isotopic fit score based on the relative mean square of the difference of an experimental mass spectrum from the theoretical isotopic pattern of a specific molecular formula) under 30 as the maximum acceptable deviation of the mass of the compound and the isotopic pattern, respectively. All the molecular formulas were obtained using the Smart Formula tool (Metaboscape v. 2022b) and the C, H, N, O, P, S, Cl, Br, I, and F elements. MSMS spectra were automatically matched with MSMS libraries: Bruker HMDB 2.0 library, MassBank of North America (MoNA) library, and NIST ver. 2020 MSMS library. For compounds annotated in Metaboscape, 2D and 3D ion images were generated. Compounds were identified by LARAPPI/CI-MSI based on acquired LC–MS data and basic metabolites from Ideom database and their representing ion images for microorganisms.

#### 3.4.3. Statistical Analysis

All metabolite datasets exported from Metaboscape v.2022b were analyzed using MetaboAnalyst 6.0 (Pang et al., 2024) [80]. Volcano plots were generated with a fold change threshold (>2 or <0.5) and a *p*-value cutoff (<0.05) to highlight metabolites showing statistically significant differences in abundance. To identify the affected metabolic pathways, pathway impact analysis was performed using the KEGG pathway library of *Bacillus subtilis subsp. Subtilis* for bacteria and the KEGG pathway library of *Aspergillus clavatus* for fungal strain, as well as the Small Molecule Pathway Database (SMPD). Pathways were ranked based on statistical *p*-values, Holm-adjusted *p*-values, and FDR from pathway topology analysis.

## 4. Conclusions

In this study, the LARAPPI/CI-MSI 3D method was used for the first time to generate ion images of bacterial and fungal metabolites in solid culture media. The LARAPPI/CI MSI mass spectrometry imaging method enabled the assessment of the spatial distribution within the colony and in the growth medium. The studied bacteria and fungi produced metabolites belonging to various chemical groups, including amino acids and derivatives, dipeptides, organic acids, fatty acids, sugars and sugar derivatives, benzene derivatives, and indoles. The LARAPPI/CI-MSI 3D method was found to be more precise than LARAPPI/CI-MSI 2D, opening new possibilities for the metabolic profiling of strains in target growth conditions/media, such as depth profiling in the absence/presence of stress factors affecting the metabolic profile. This innovative method can be used for the rapid screening of the biotechnological potential of environmental strains, facilitating the search for industrially valuable biomolecules.

## Figures and Tables

**Table 1:** LARAPPI/CI-MSI 2D ion images of dipeptides and their derivatives produced from the tested microbial cultures.

Type	Name (Tested Microorganisms)	Ion Image
Pro-Leu	(left: *F. graminarum*; right: *P. amylolyticus*)	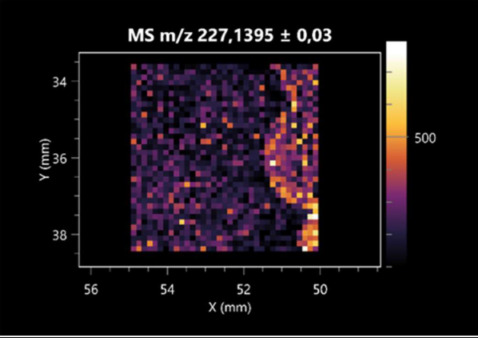
PyroGlu-Ala	(left: *B. cereus*; right: *F. graminarum*)	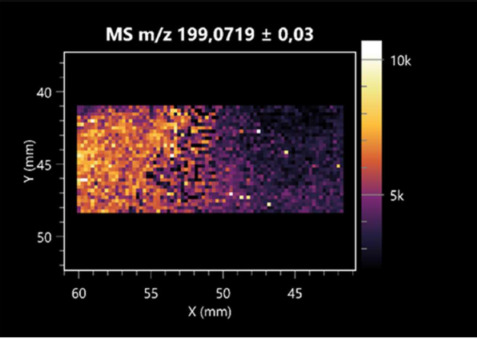
Pro-Pro	(left: *B. cereus*; right: *F. graminarum*)	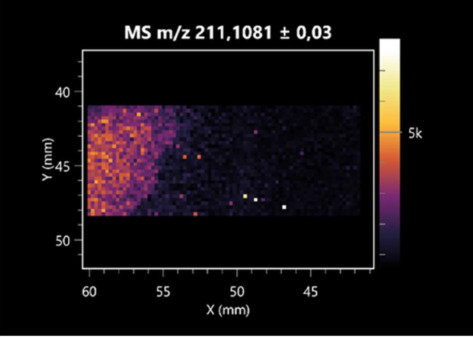
Pro-Val	(left: *B. cereus*; right: *F. graminarum*)	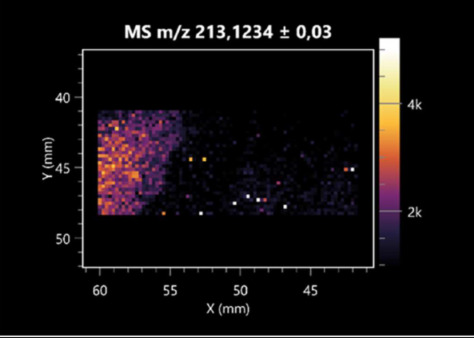
Leu-Pro	(left: *B. cereus*; right: *F. graminarum*)	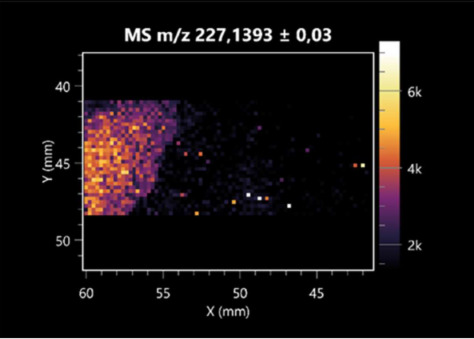
Pro-Asn	(left: *B. cereus*; right: *F. graminarum*)	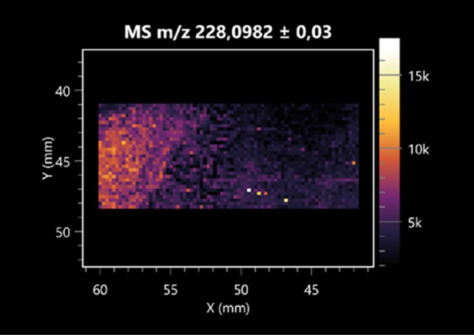
Asp-Leu	(left: *B. cereus*; right: *F. graminarum*)	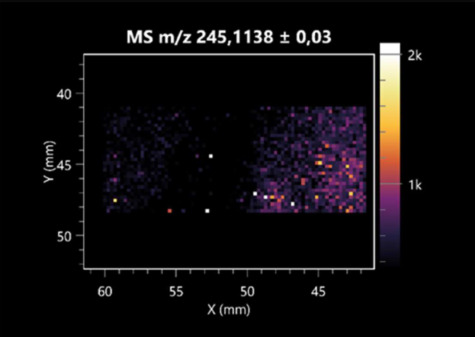

**Table 2:** LARAPPI/CI-MSI 2D ion images of amino acids and their derivatives produced from the tested microbial cultures.

Name (Tested Microorganisms)	Ion Image
L-glutamine (left: *F. graminarum*; right: *P. amylolyticus*)	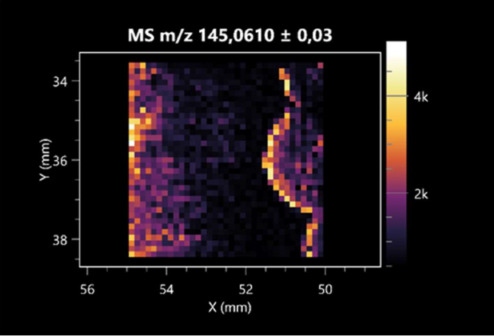
L-glutamic acid (left: *F. graminarum*; right: *P. amylolyticus*)	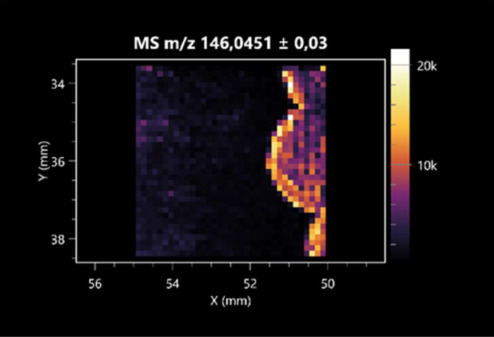
L-arginine (left: *F. graminarum*; right: *P. amylolyticus*)	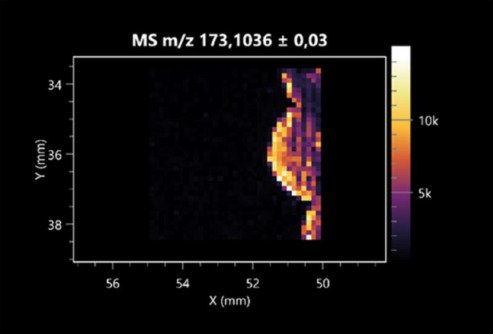
Diaminopimelic acid (left: *F. graminarum*; right: *P. amylolyticus*)	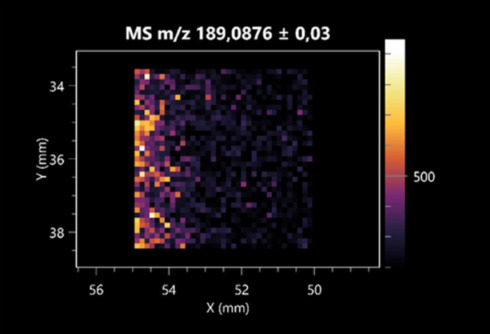
L-proline (left: *B. cereus* right: *F. graminarum*)	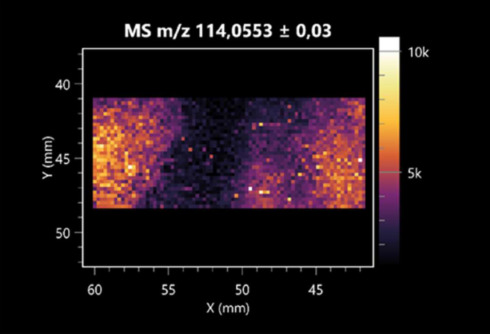
L-aspartic acid (left: *B. cereus*; right: *F. graminarum*)	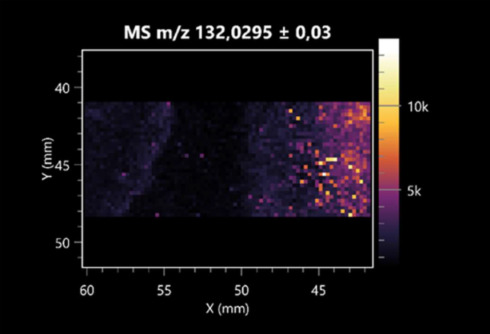
Citrulline (left: *B. cereus*; right: *F. graminarum*)	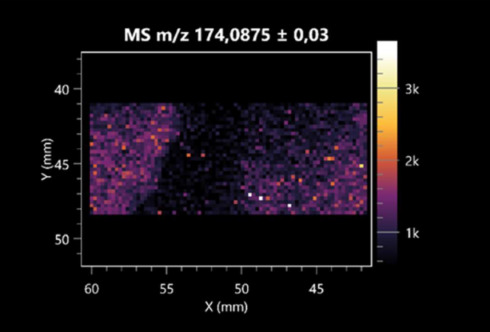
L-tryptophan (left: *B. cereus*; right: *F. graminarum*)	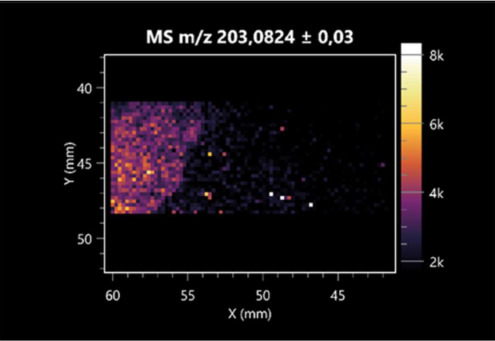
*N*-acetylglycine (left: *F. graminarum;* right: *P. amylolyticus*)	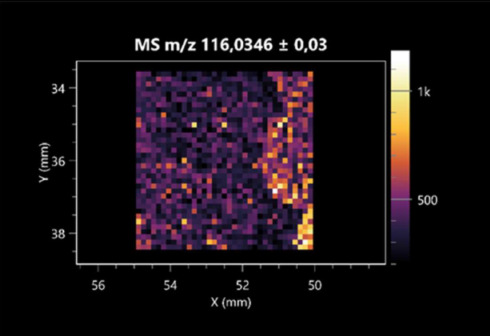
Creatine (left: *B. cereus*; right: *F. graminarum*)	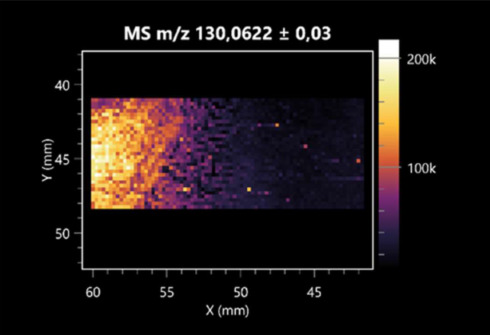
Aminoadipic acid (left: *B. cereus*; right: *F. graminarum*)	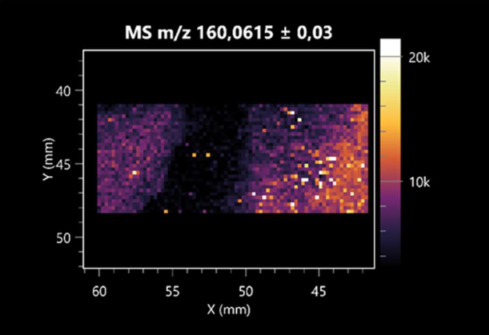
4-acetamidobutanoic acid (left: *B. cereus*; right: *F. graminarum*)	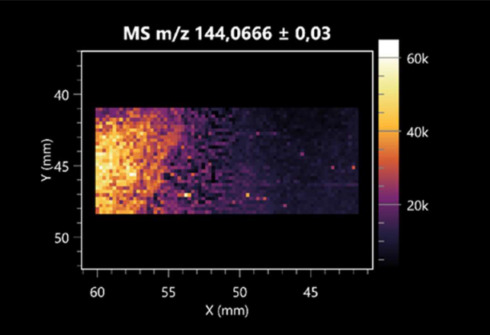

**Table 3:** LARAPPI/CI-MSI 2D ion images of organic acids and their derivatives produced from the tested microbial cultures.

Name	Tested Microorganisms	Ion Image
Pyrrolidonecarboxylic acid	(left: *F. graminarum*; right: *P. amylolyticus*)	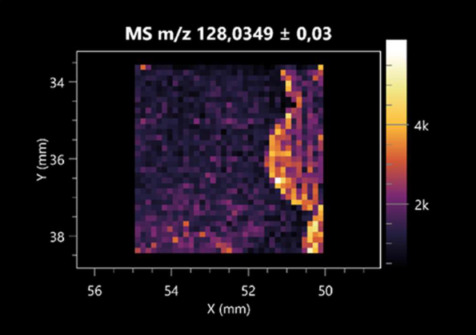
Malic acid	(left: *F. graminarum*; right: *P. amylolyticus*)	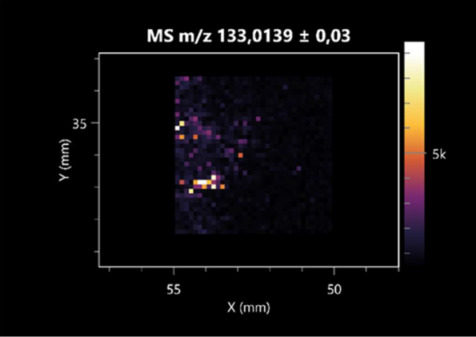
Citric acid	(left: *B. cereus*; right: *F. graminarum*)	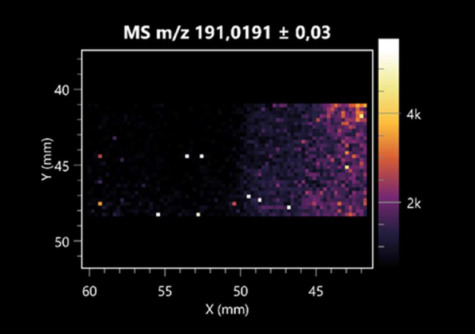
Sebacic acid	(left: *B. cereus*; right: *F. graminarum*)	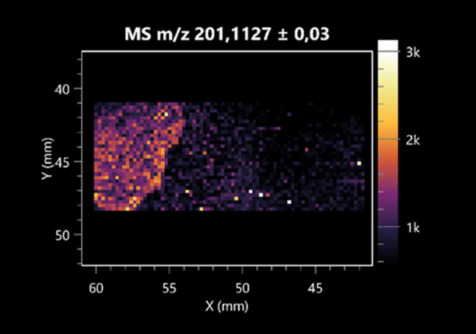
Pantothenic acid	(left: *B. cereus*; right: *F. graminarum*)	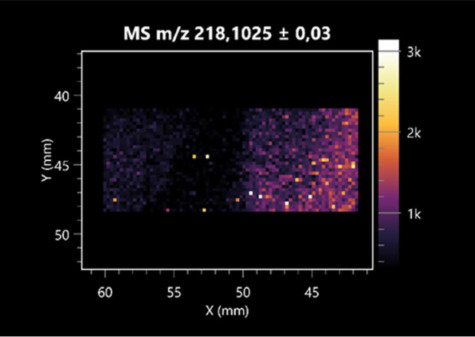
Propionic acid	(left: *B. cereus*; right: *F. graminarum*)	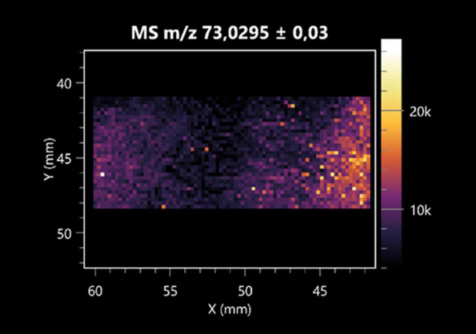
Lactic acid	(left: *B. cereus*; right: *F. graminarum*)	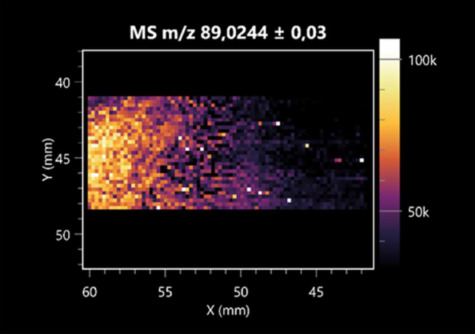
2-furoic acid	(left: *B. cereus*; right: *F. graminarum*)	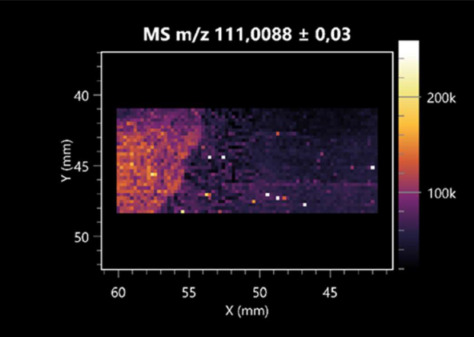
Fumaric acid	(left: *B. cereus*; right: *F. graminarum*)	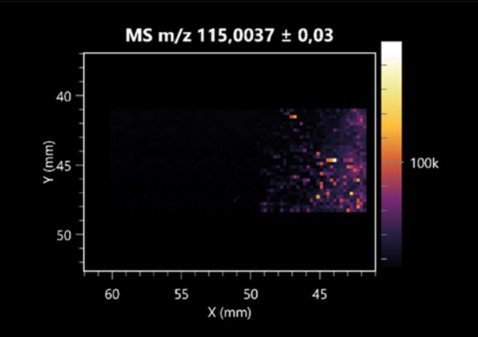
Succinic acid	(left: *B. cereus*; right: *F. graminarum*)	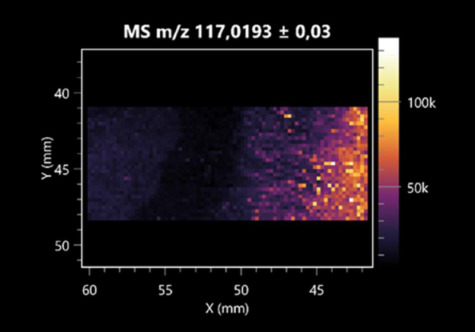
Hydroxypropanedioic acid	(left: *B. cereus*; right: *F. graminarum*)	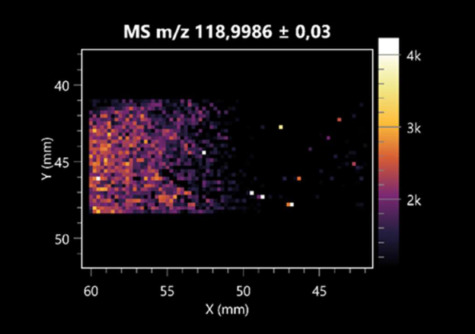
Dihydroxyfumaric acid	(left: *B. cereus*; right: *F. graminarum*)	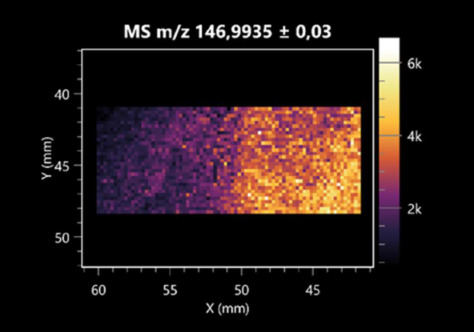
2-Aminobenzoic acid	(left: *B. cereus* right: *F. graminarum*)	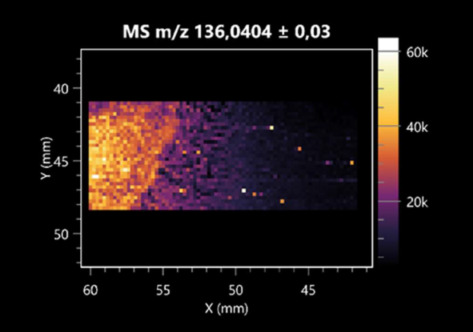
Xanthurenic acid	(left: *B. cereus*; right: *F. graminarum*)	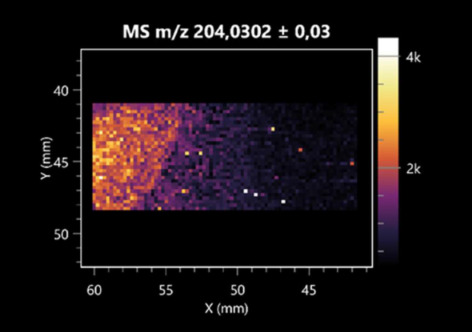
Kynurenic acid	(left: *B. cereus*; right: *F. graminarum*)	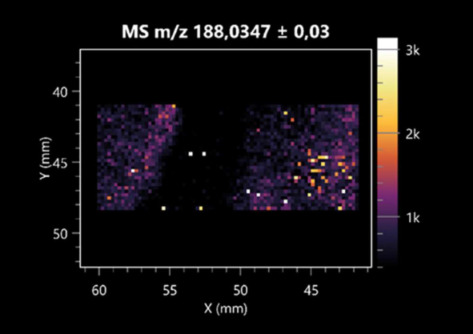

**Table 4:** LARAPPI/CI-MSI 2D ion images of fatty acids and their derivatives produced from the tested microbial cultures.

Name	Tested Microorganisms	Ion Image
Azelaic acid	(left: *F. graminarum*; right: *P. amylolyticus*)	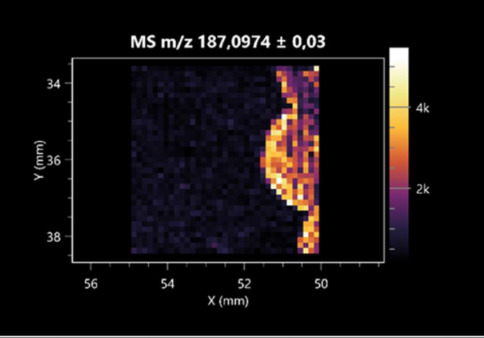
	(left: *B. cereus*; right: *F. graminarum*)	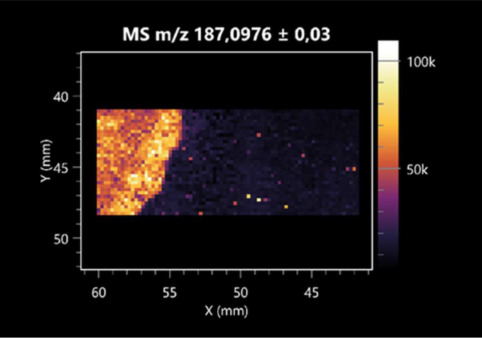
Elaidic acid	(left: *B. cereus*; right: *F. graminarum*)	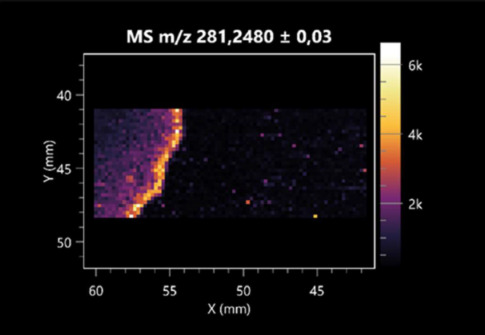
Linolenic acid	(left: *B. cereus*; right: *F. graminarum*)	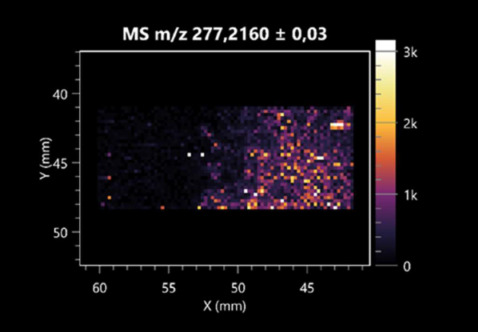
Linoleic acid	(left: *B. cereus*; right: *F. graminarum*)	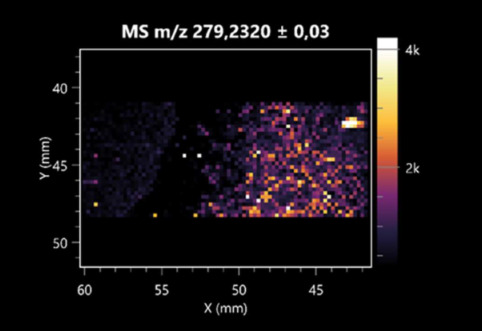
Pentadecanoic acid	(left: *B. cereus*; right: *F. graminarum*)	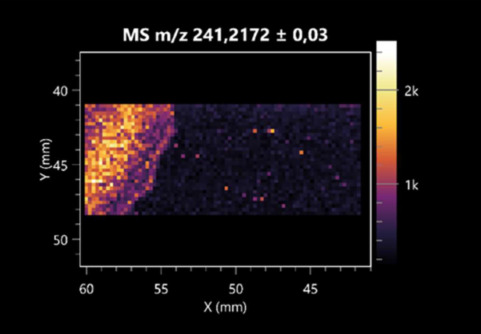
Valeric acid	(left: *B. cereus*; right: *F. graminarum*)	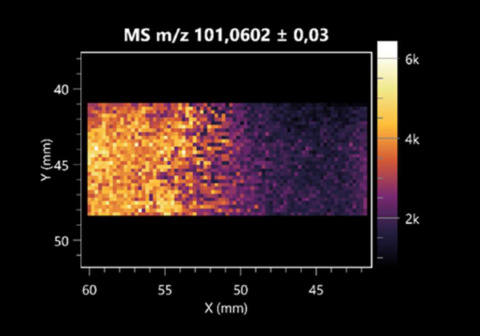

**Table 5:** LARAPPI/CI-MSI 2D ion images of sugars and their derivatives produced from the tested microbial cultures.

Name	Tested Microorganisms	Ion Image
Ribitol	(left: *F. graminarum*; right: *P. amylolyticus*)	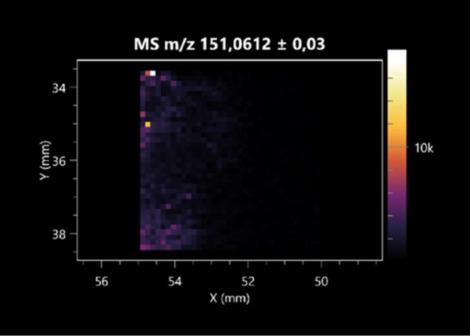
Sorbitol	(left: *F. graminarum*; right: *P. amylolyticus*)	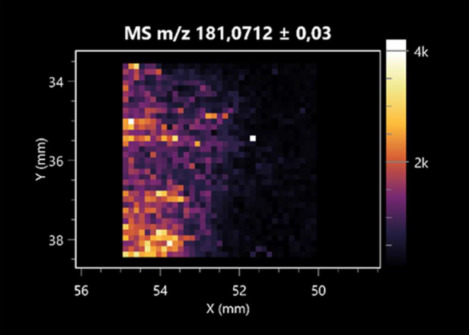
2-amino-2-deoxy-D-mannitol	(left: *B. cereus*; right: *F. graminarum*)	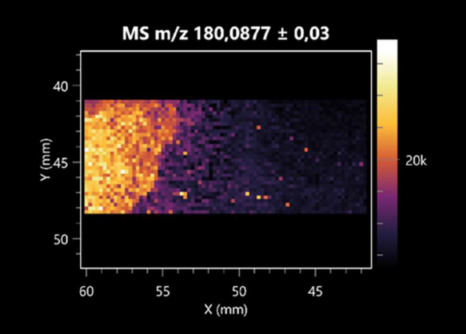
4-amino-4-deoxy-L-arabinose	(left: *B. cereus*; right: *F. graminarum*)	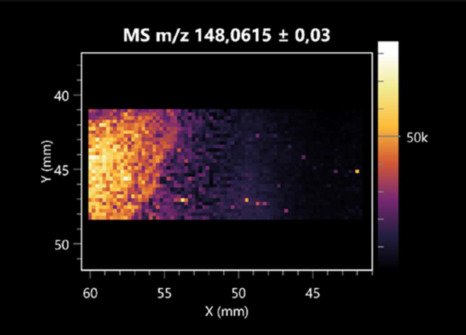
Erythronic acid	(left: *B. cereus*; right: *F. graminarum*)	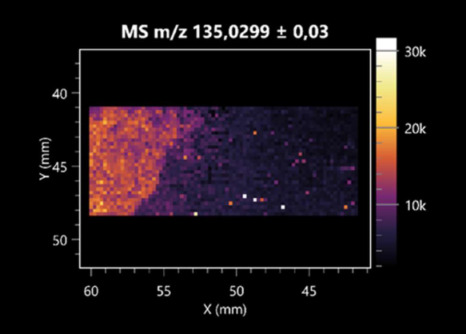
Deoxyribose 5-phosphate	(left: *B. cereus*; right: *F. graminarum*)	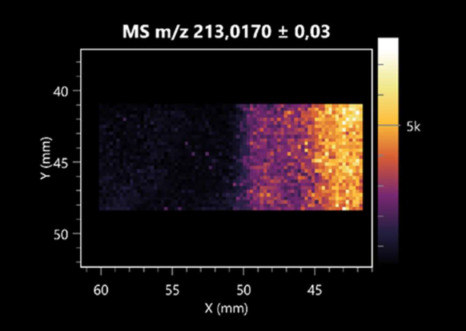
*N*-acetylmannosamine	(left: *B. cereus*; right: *F. graminarum*)	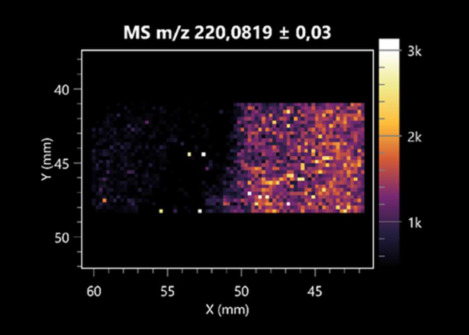
Trehalose	(left: *B. cereus*; right: *F. graminarum*)	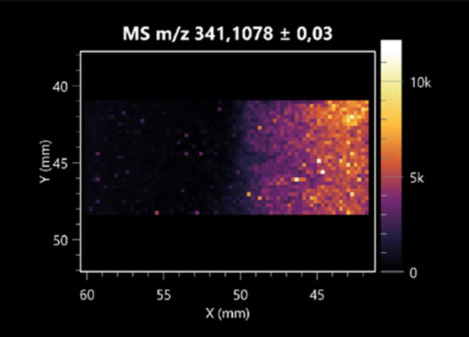

**Table 6:** LARAPPI/CI-MSI 3D ion images of bacterial and fungal metabolites produced from *Paenibacillus amylolyticus* (right) and *Fusarium graminarum* (left) cultures.

Compound		Ion Images
Amino acids and derivatives	L-glutamine	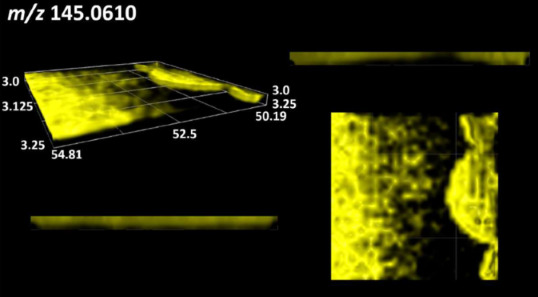
	L-glutamic acid	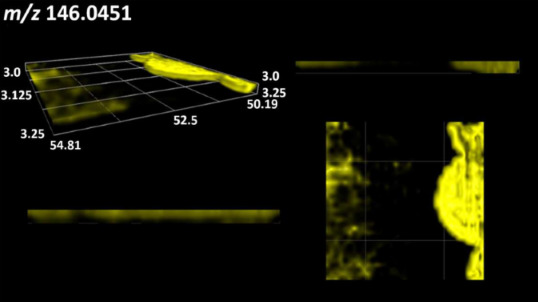
	L-arginine	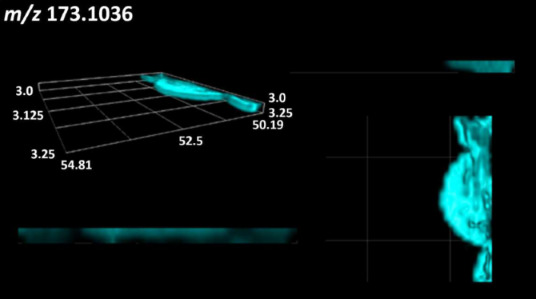
	*N*-acetylglycine	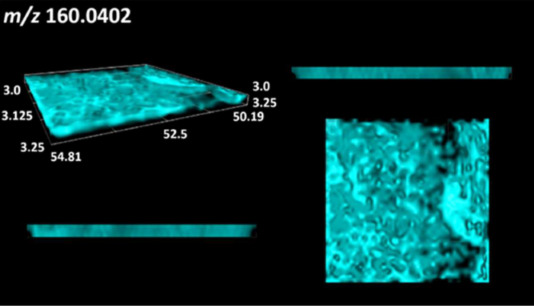
	Diaminopimelic acid	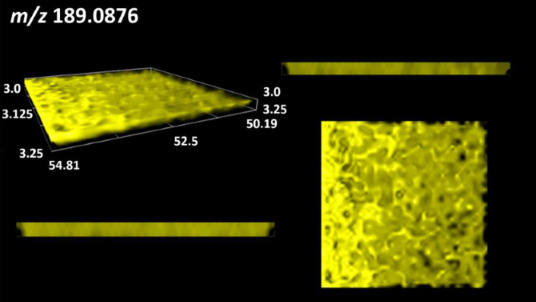
Organic acids	Pyrrolidonecarboxylic acid	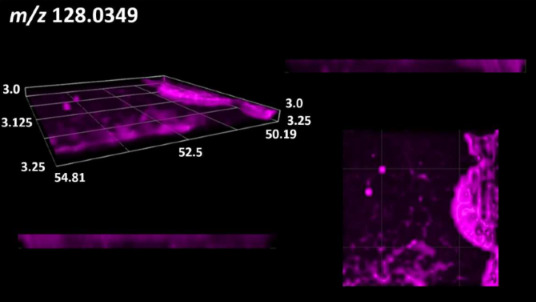
	Malic acid	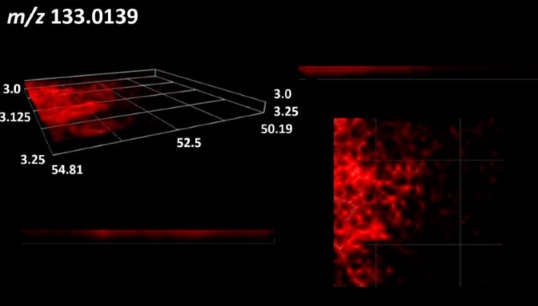
Dipeptides	Pro-Leu	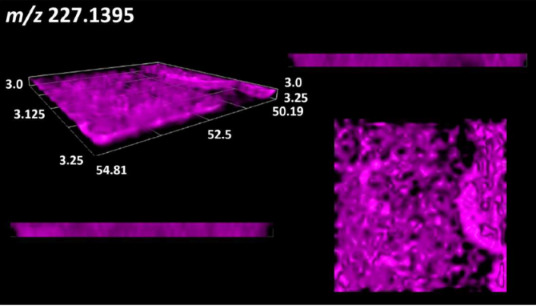
Fatty acids	Azelaic acid	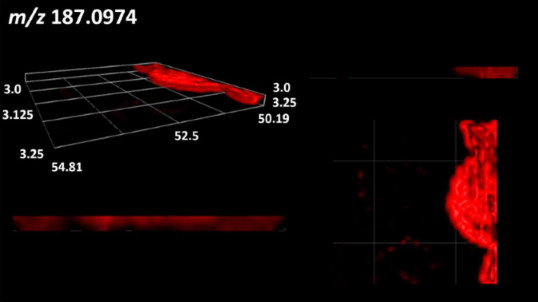
Sugars and sugar derivatives	Ribitol	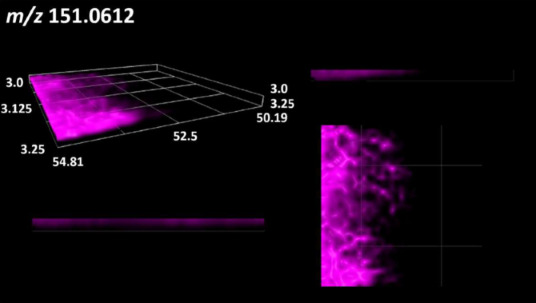
	Sorbitol	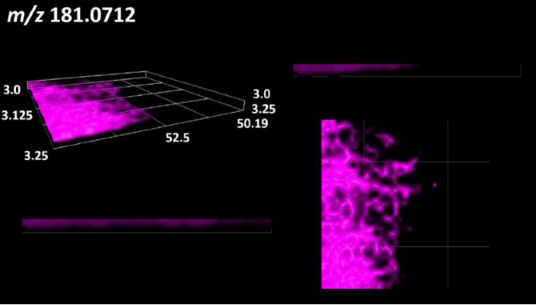
	Deoxyguanosine	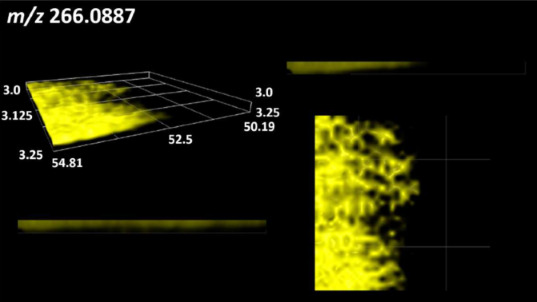
Benzene derivatives	*N*-methylbenzamide	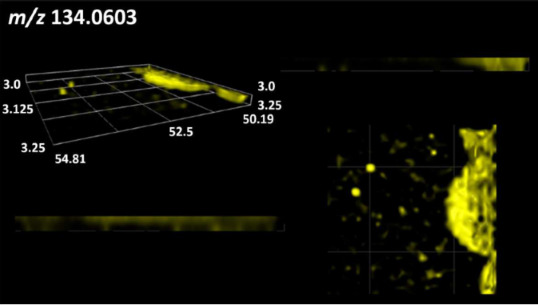
Indoles	Indole-3-carboxylic acid	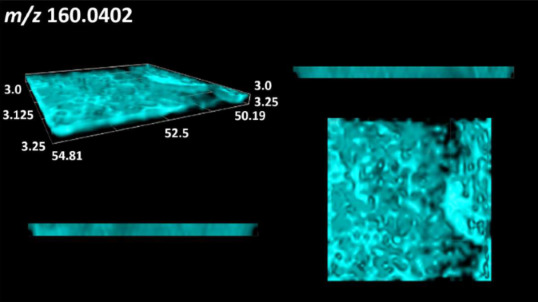

## Data Availability

The datasets generated during and/or analyzed during the current study are available from the corresponding author upon request and in the RepOD open data repository (DOI: https://doi.org/10.18150/YCUODU).

## Supplementary Materials

The following supporting information can be downloaded at https://www.mdpi.com/article/10.3390/molecules30061317/s1: Table S1. LARAPPI/CI-MSI 2D ion images of metabolites from the tested microbial culture; Table S2. LARAPPI/CI-MSI 3D ion images of bacterial and fungal metabolites from *Paenibacillus amylolyticus* (right) and *Fusarium graminarum* (left) cultures; Table S3. Pathway enrichment analysis of metabolites in *Paenibacillus amylolyticus*, highlighting the matched pathways, key metabolites, and statistical significance for each metabolic pathway; Table S4. Pathway enrichment analysis of metabolites in *Bacillus cereus*, highlighting the matched pathways, key metabolites, and statistical significance for each metabolic pathway; Table S5. Pathway enrichment analysis of metabolites in *Fusarium graminarum*, highlighting the matched pathways, key metabolites, and statistical significance for each metabolic pathway; Table S6. Enrichment analysis of main-class chemical structures in *Paenibacillus amylolyticus*; Table S7. Enrichment analysis of main-class chemical structures in *Bacillus cereus*; Table S8. Enrichment analysis of main-class chemical structures in *Fusarium graminarum*; Table S9. LC–MS data supporting generated LARAPPI/CI-MSI 2D and LARAPPI/CI-MSI 3D ion images. Figure S1. MS spectra of top ablation level of 3D MSI. Upper spectrum represents one voxel for *P. amylolyticus* area, bottom for *F. graminarum* medium area. Figure S2. Volcano plot displaying the most discriminating compounds between *Paenibacillus amylolyticus* and *Fusarium graminearum*, identified from microbial extracts analyzed using UHPLC–HRMS and visualized by LARAPPI/CI-MSI 3D. Figure S3. Volcano plot displaying the most discriminating compounds between Bacillus cereus and Fusarium graminearum, identified from microbial extracts analyzed using UHPLC–HRMS and visualized by LARAPPI/CI-MSI 3D. Figure S4. Box plots of the most discriminating compounds between *Paenibacillus amylolyticus* (P.A.) and *Fusarium graminearum* (F.G.) identified from microbial extracts analyzed using UHPLC–HRMS and visualized by 3D LARAPPI/CI-MSI. Figure S5. Box plots of the most discriminating compounds between *Fusarium graminearum* (F.G.) and *Bacillus cereus* (B.C.), identified from microbial extracts analyzed using UHPLC–HRMS and visualized by 3D LARAPPI/CI-MSI.

## Abbreviations

The following abbreviations are used in this manuscript:

UHPLC	Ultra-high-performance liquid chromatography
UHRMS	Ultra-high-resolution mass spectrometry
LARAPPI/CI	Laser ablation remote atmospheric pressure photoionization/chemical ionization
MSI	Mass spectrometry imaging
APCI	Atmospheric pressure chemical ionization
ESI	Electrospray ionization
