# Supplementary material for: Direct 3D Mass Spectrometry Imaging Analysis of Environmental Microorganisms
Source: Molecules. 2025 Mar 14;30(6):1317. doi: 10.3390/molecules30061317 (PMC11946574; doi:10.3390/molecules30061317)
Supplement: Supplementary file 1 [file molecules-30-01317-s001.zip › Figure S1-S5.pdf]

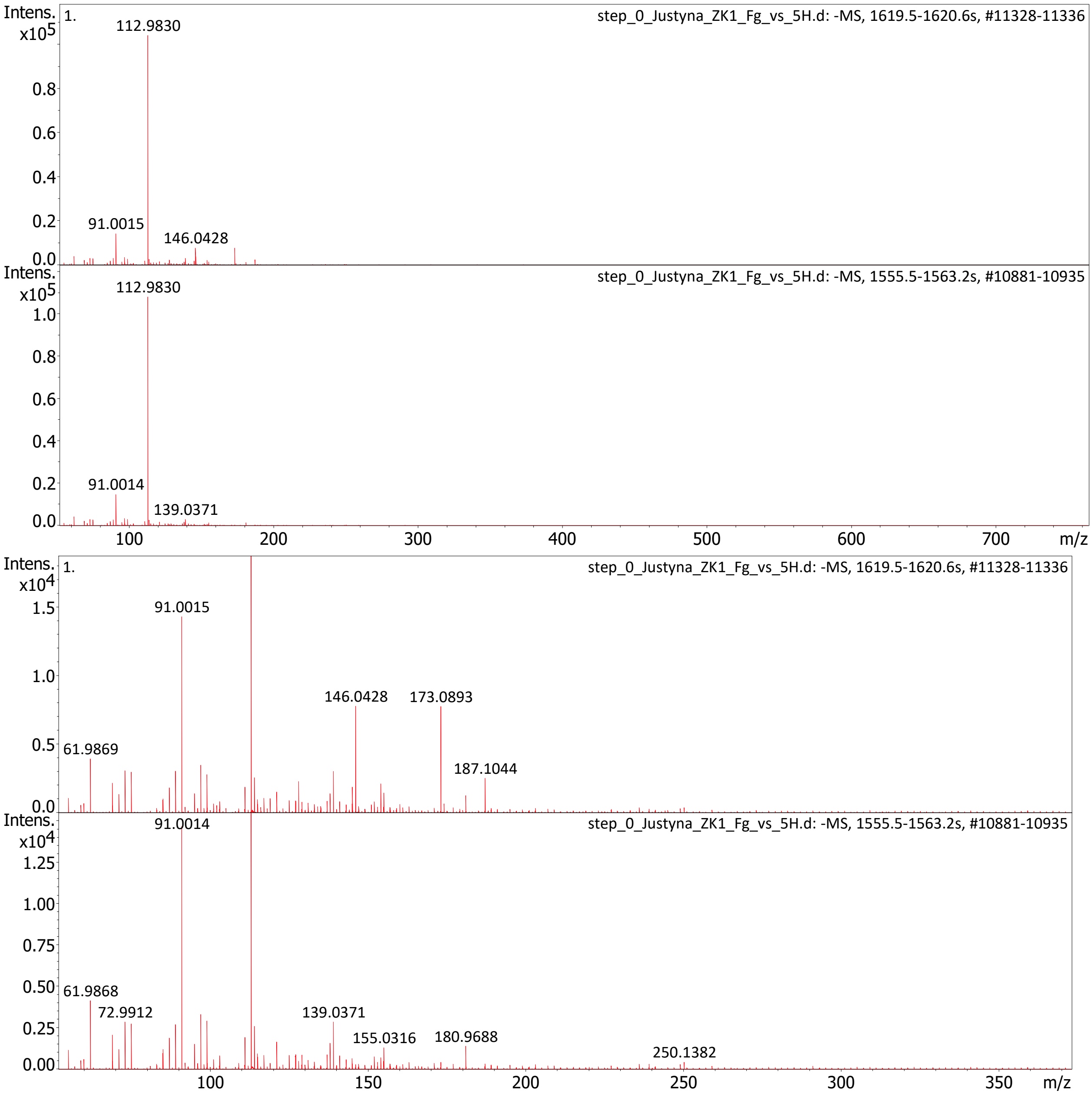

Figure S1. MS spectra of top ablation level of 3D MSI. Upper spectrum represents one voxel for *P. amylolyticus* area, bottom for *F. graminarum* agar medium area.

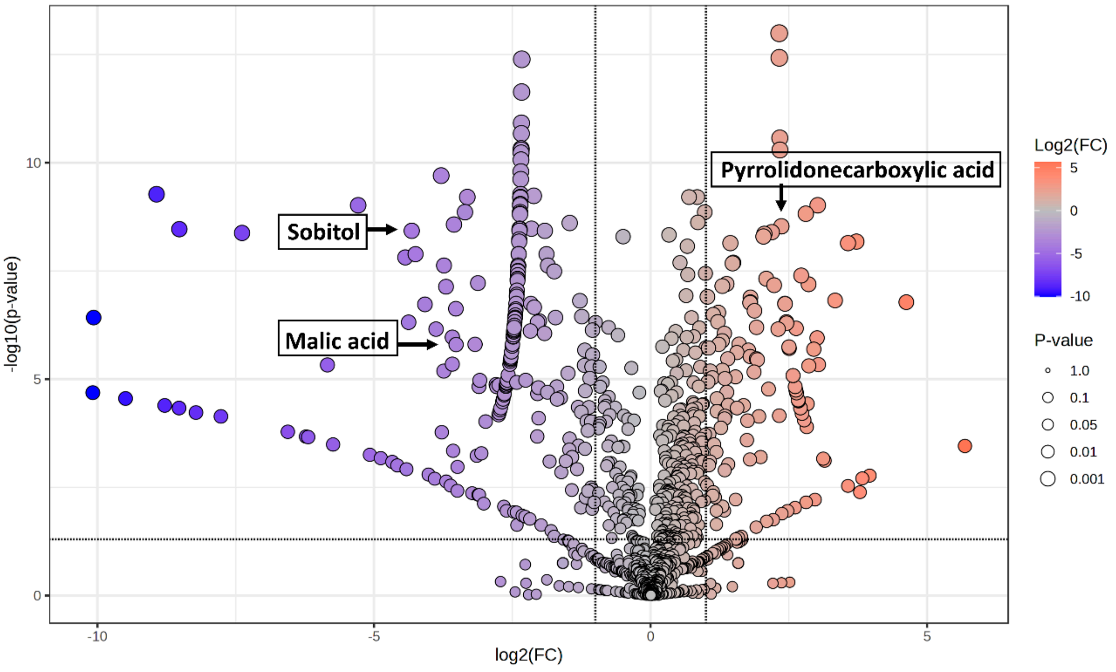

Figure S2. Volcano plot displaying the most discriminating compounds between *Paenibacillus amylolyticus* and *Fusarium graminearum*, identified from microbial extracts analyzed using UHPLC-HRMS and visualized by LARAPPI/CI-MSI 3D. Red points denote compounds significantly more abundant in *P. amylolyticus*, blue points denote compounds significantly more abundant in F. graminearum, and gray points represent compounds below the significance threshold.

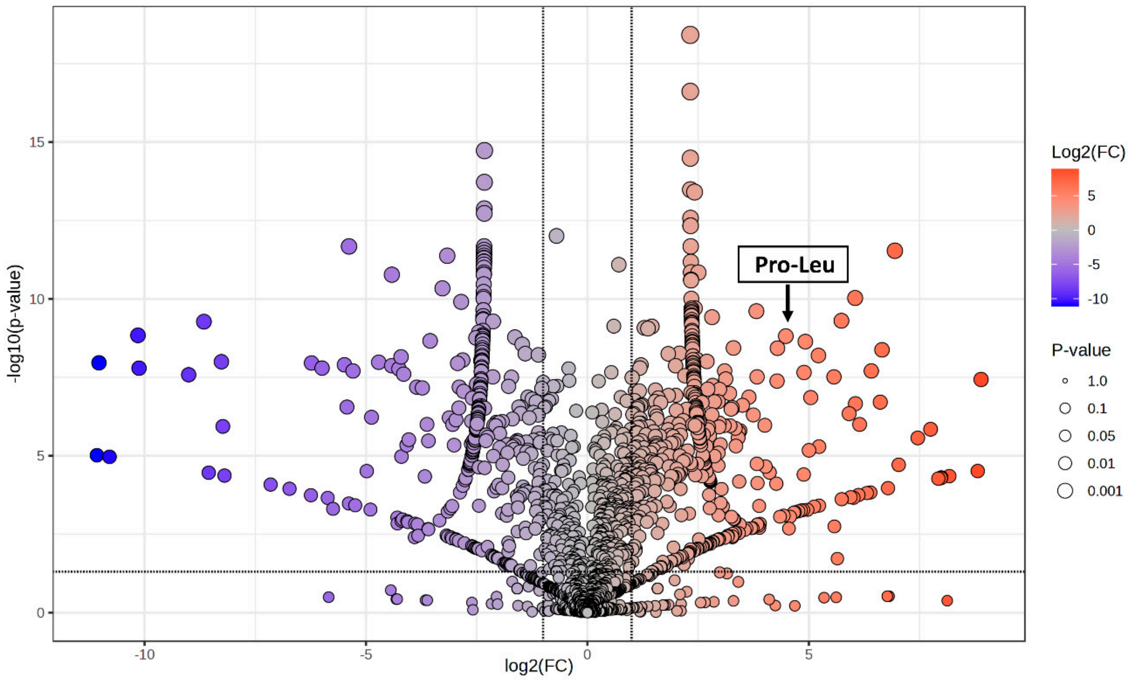

Figure S3. Volcano plot displaying the most discriminating compounds between *Bacillus cereus* and *Fusarium graminearum*, identified from microbial extracts analyzed using UHPLC-HRMS and visualized by LARAPPI/CI-MSI 3D. Red points denote compounds significantly more abundant in *B. cereus*, blue

points denote compounds significantly more abundant in *F. graminearum*, and gray points represent compounds below the significance threshold.

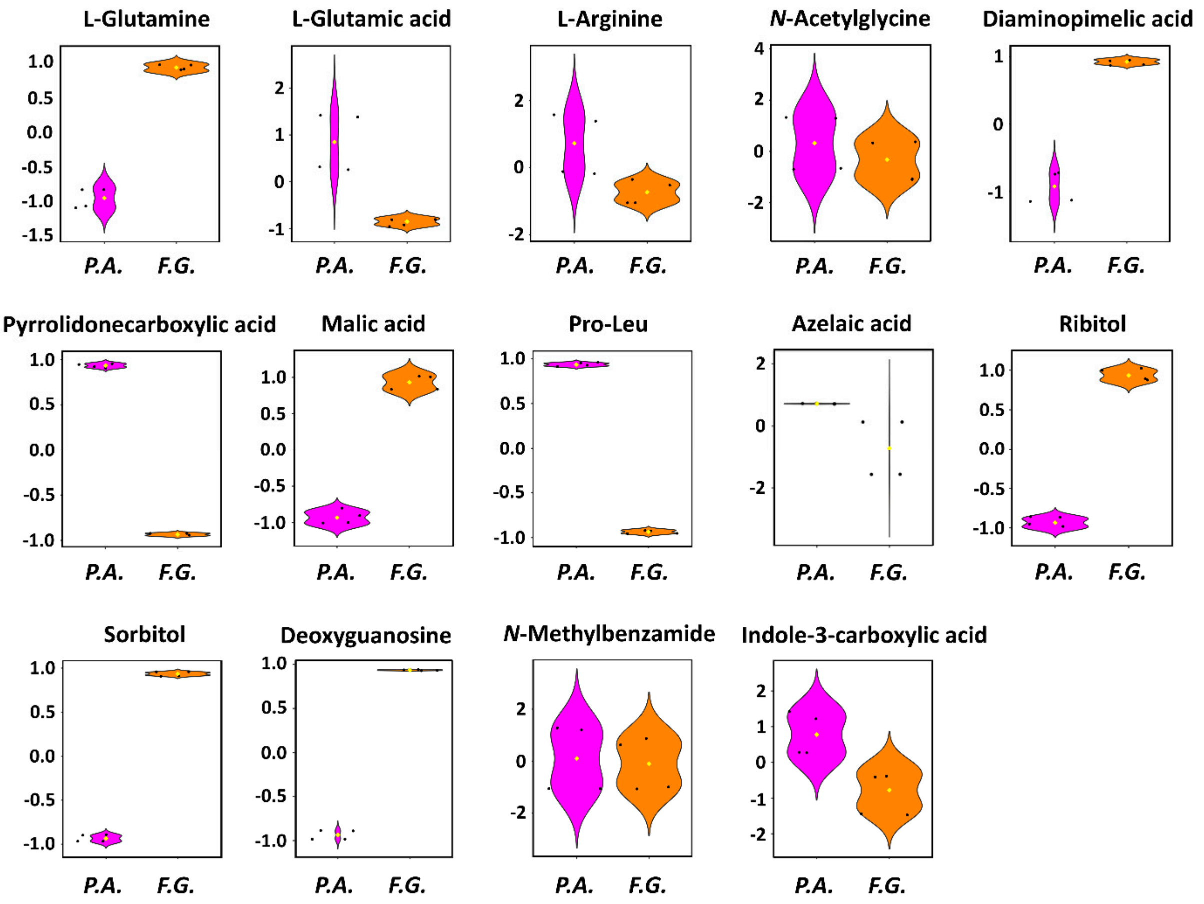

Figure S4. Box plots of the most discriminating compounds between *Paenibacillus amylolyticus* (*P.A.*) and *Fusarium graminearum* (*F.G.*) identified from microbial extracts analyzed using UHPLC-HRMS and visualized by 3D LARAPPI/CI-MSI. Each subplot shows the distribution of a particular metabolite in *P.A.* (purple) and *F.G.* (orange), highlighting the differences in abundance between these two organisms.

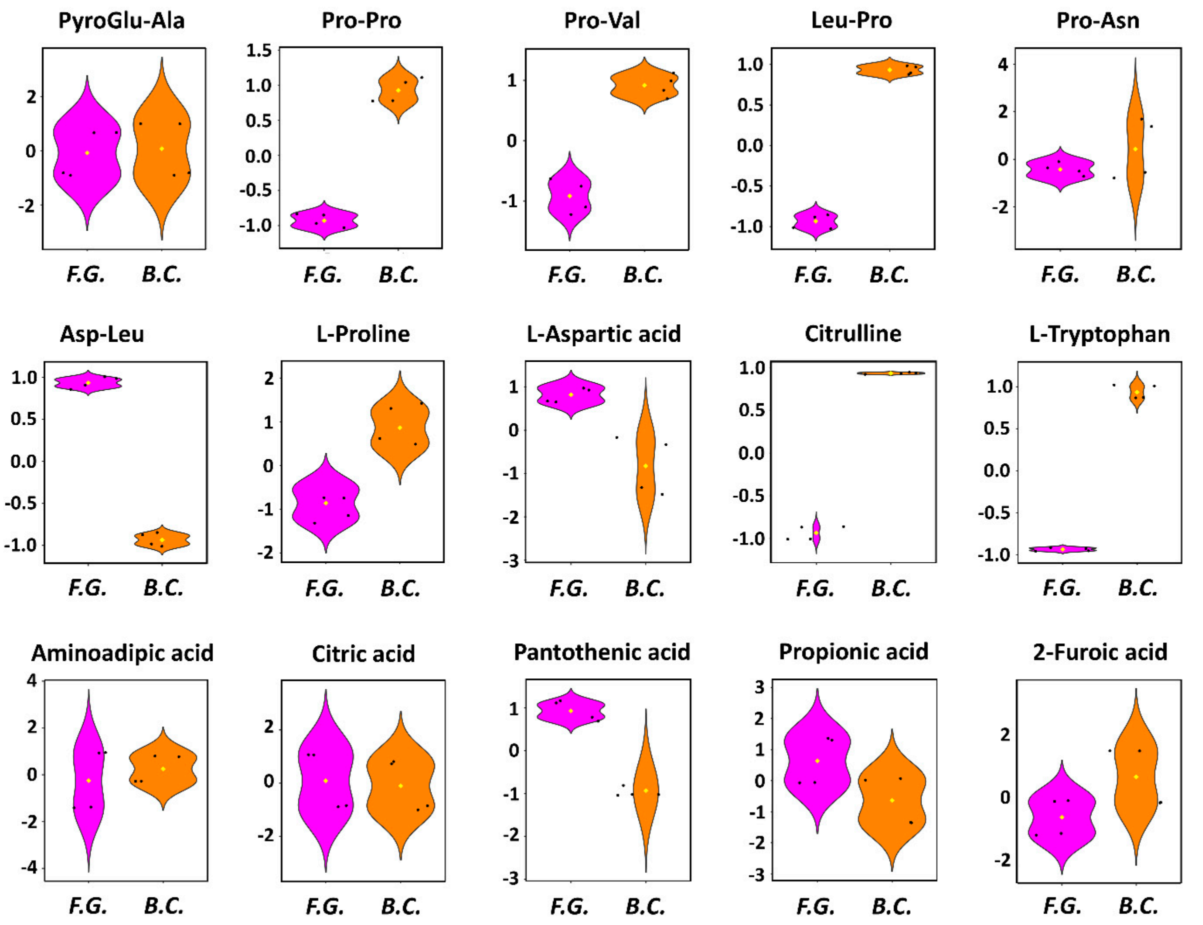

Figure S5. Box plots of the most discriminating compounds between *Fusarium graminearum* (*F.G.*) and *Bacillus cereus* (*B.C.*), identified from microbial extracts analyzed using UHPLC-HRMS and visualized by 3D LARAPPI/CI-MSI. Each subplot shows the distribution of a particular metabolite in *F.G.* (purple) and *B.C.* (orange), highlighting the differences in abundance between these two organisms.
