# Supplementary material for: Direct 3D Mass Spectrometry Imaging Analysis of Environmental Microorganisms
Source: Molecules. 2025 Mar 14;30(6):1317. doi: 10.3390/molecules30061317 (PMC11946574; doi:10.3390/molecules30061317)
Supplement: Supplementary file 1 [file molecules-30-01317-s001.zip › Table S1_.pdf]

Table S1. LARAPPI/CI-MSI 2D ion images of metabolites from tested microbial culture

Type	Name<br>(tested microorgnisms)	Ion image
**Amino acids and derivatives**	D-α -Aminobutyric acid<br><br>(left: *F. graminarum*<br>right: *P. xylanexedens*)	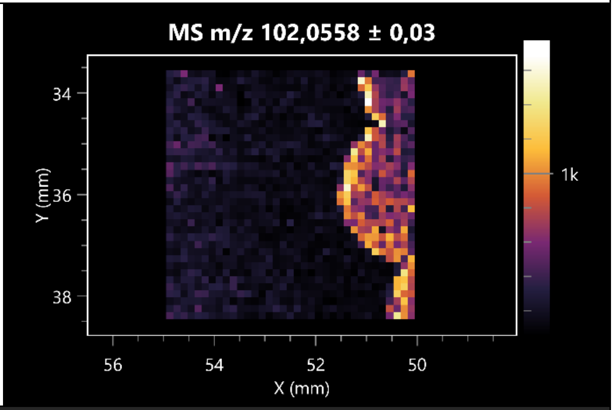
	L- α -aminobutyric acid<br><br>(left: *B. cereus*<br>right: *F. graminarum*)	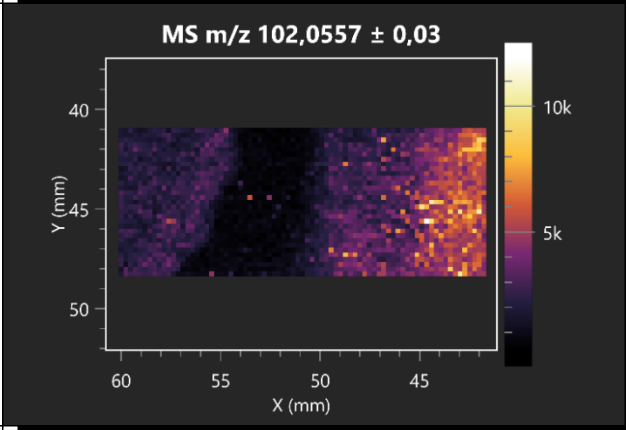
	N-Acetyl-L-alanine<br><br>(left: *F. graminarum*<br>right: *P. xylanexedens*)	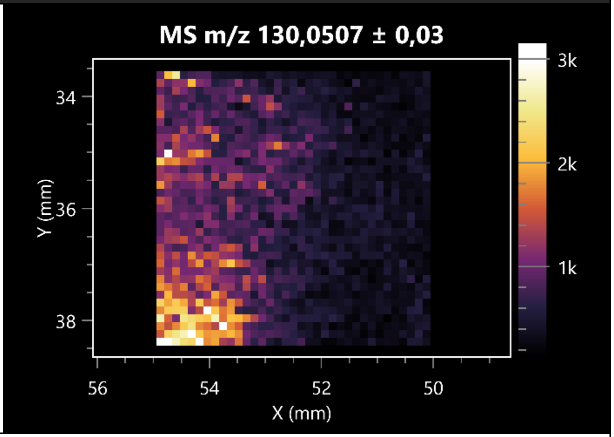
	O-Phospho-4-hydroxy-L-threonine<br><br>(left: *B. cereus*<br>right: *F. graminarum*)	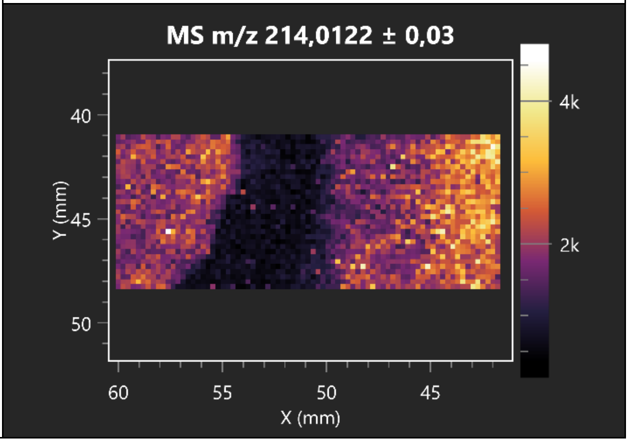

	O-Phosphothreonine<br><br>(left: *B. cereus*<br>right: *F. graminarum*)	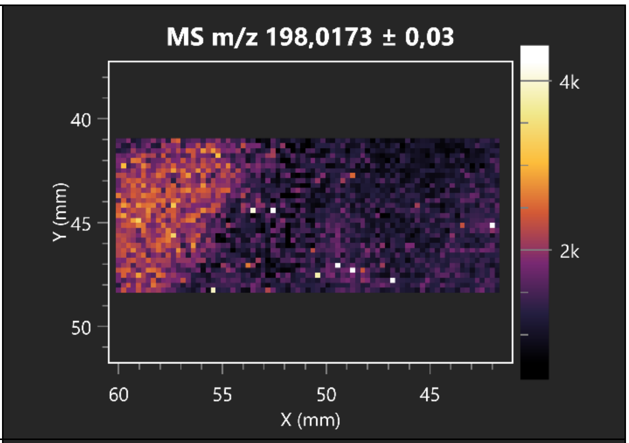
	4-Acetamidobutanoic acid<br><br>(left: *B. cereus*<br>right: *F. graminarum*)	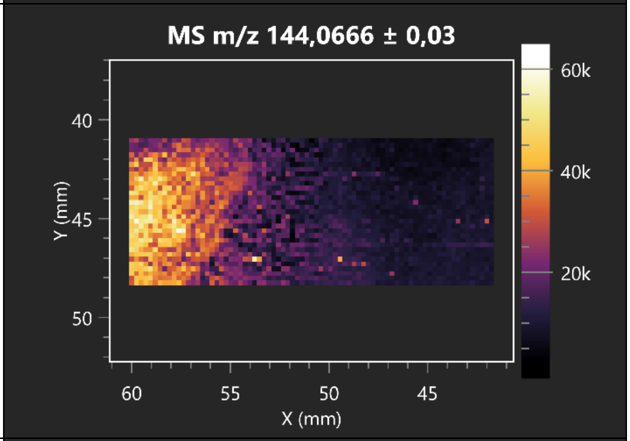
**Organic acids and derivatives**	Orthanilate<br><br>(left: *B. cereus*<br>right: *F. graminarum*)	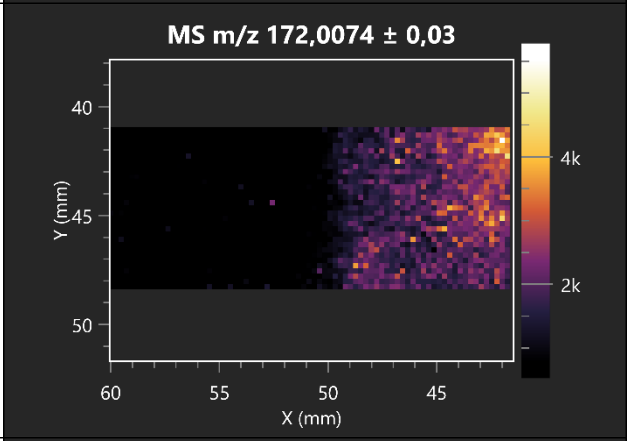
	Pantoic acid<br><br>(left: *B. cereus*<br>right: *F. graminarum*)	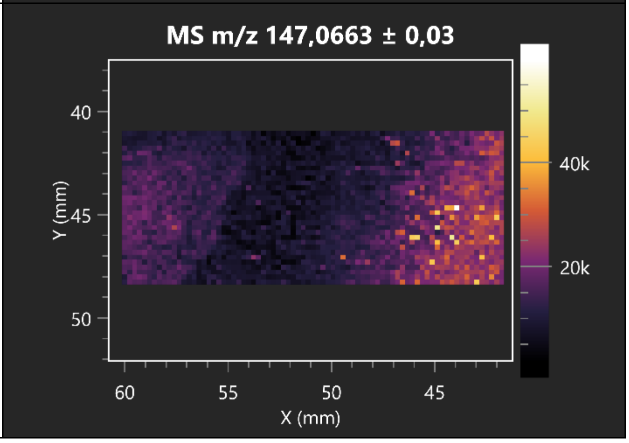

	3-Phospho-D-erythronic acid (left: *B. cereus* right: *F. graminarum*)	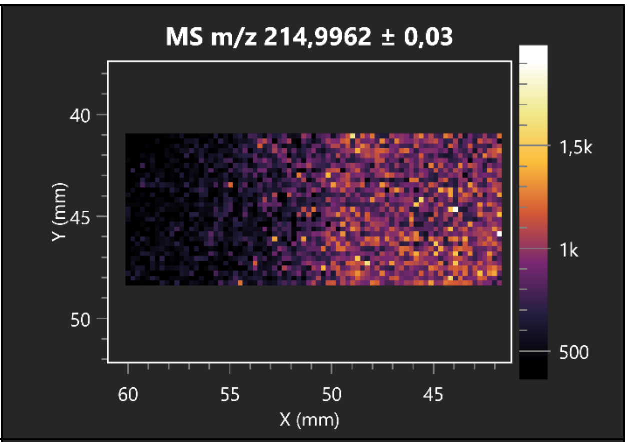
	Vanillylmandelic acid<br><br>(left: *B. cereus* right: *F. graminarum*)	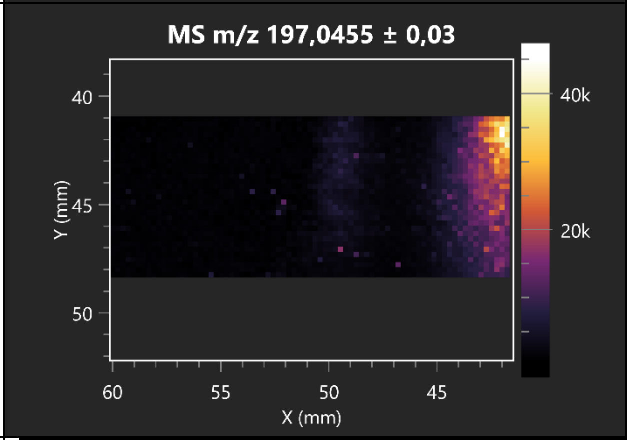
**Sugar and their derivatives**	Deoxyguanosine<br><br>(left: *F. graminarum* right: *P. xylanexedens*)	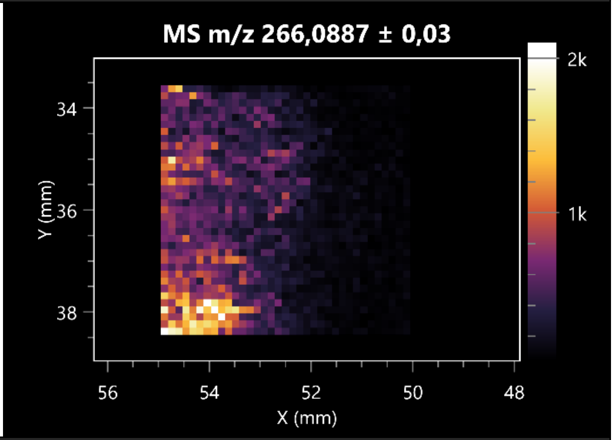
	Erythritol 4-phosphate (left: *B. cereus* right: *F. graminarum*)	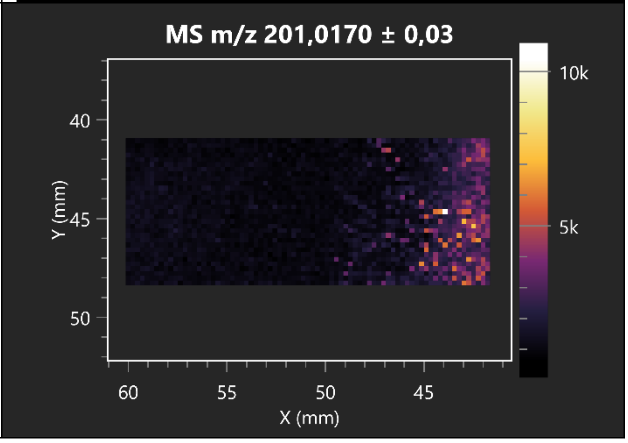

**Alcohols**	1-Heptanol<br>(left: *B. cereus*<br>right: *F. graminarum*)	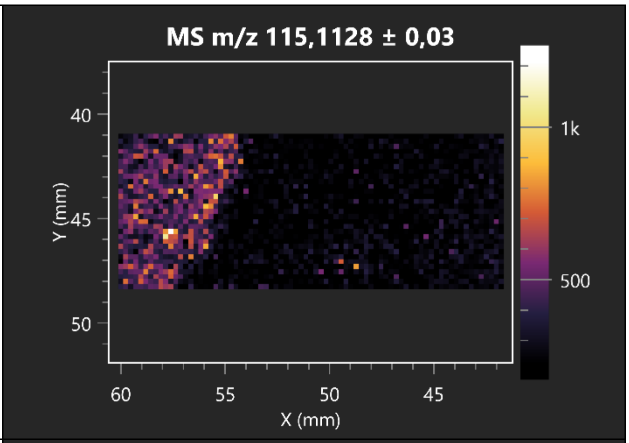
	1-Nonanol<br>(left: *B. cereus*<br>right: *F. graminarum*)	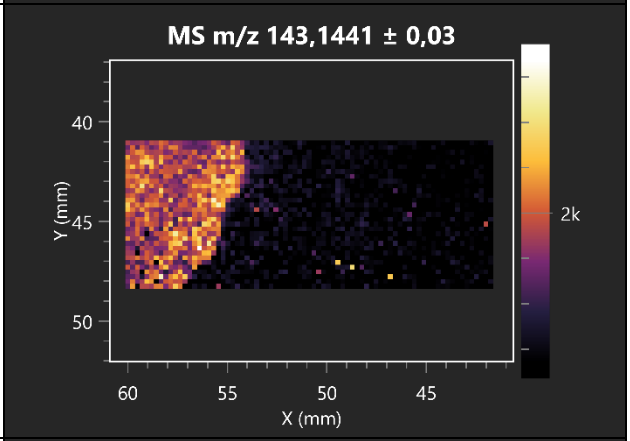
	2,3-Butanediol<br>(left: *B. cereus*<br>right: *F. graminarum*)	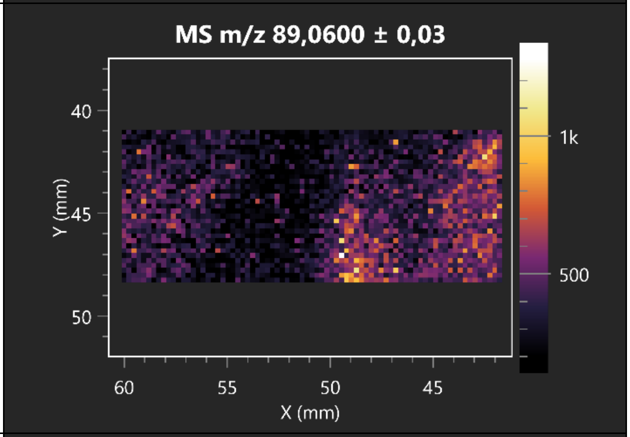
**Indoles**	Indole<br>(left: *B. cereus*<br>right: *F. graminarum*)	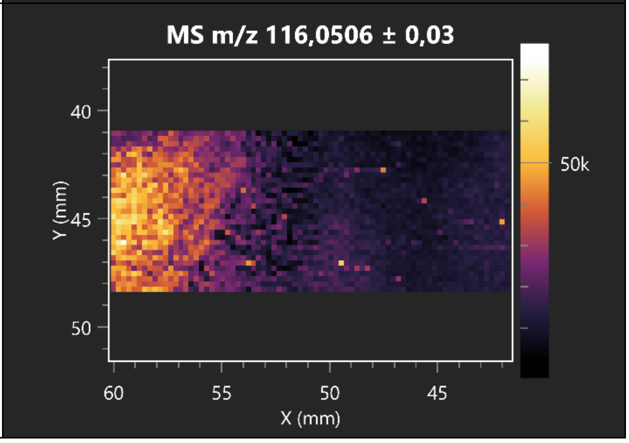

	N-Isobutyl-2-(2-methyl-1H-indol-1-yl)acetamide<br><br>(left: *B. cereus*<br>right: *F. graminarum*)	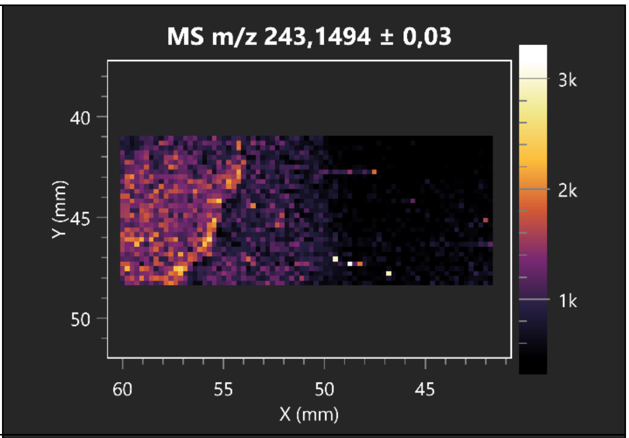
	Indole-5,6-quinone<br><br>(left: *B. cereus*<br>right: *F. graminarum*)	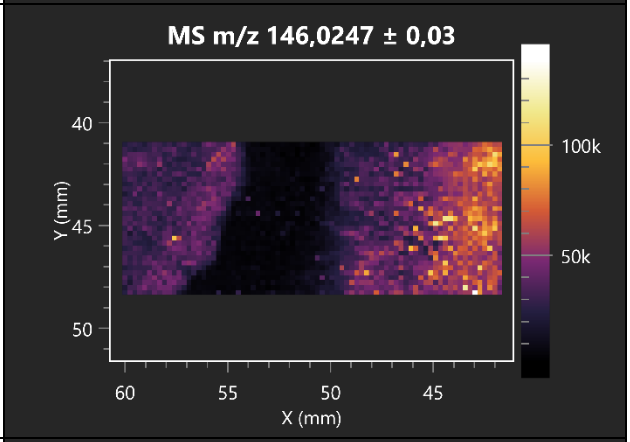
	Tryptamine<br><br>(left: *B. cereus*<br>right: *F. graminarum*)	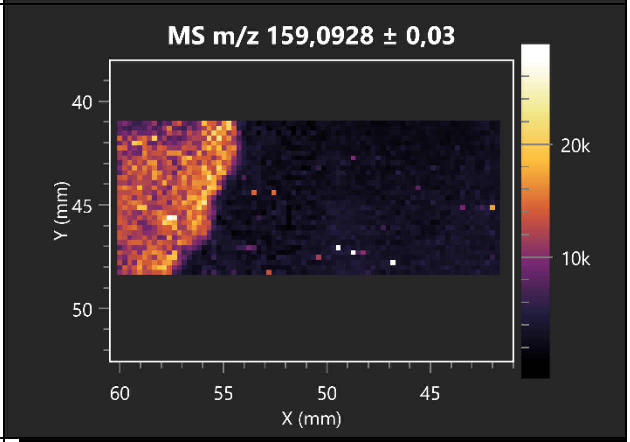
	Indole-3-carboxylic acid<br><br>(left: *F. graminarum* right: *P. xylanexedens*)	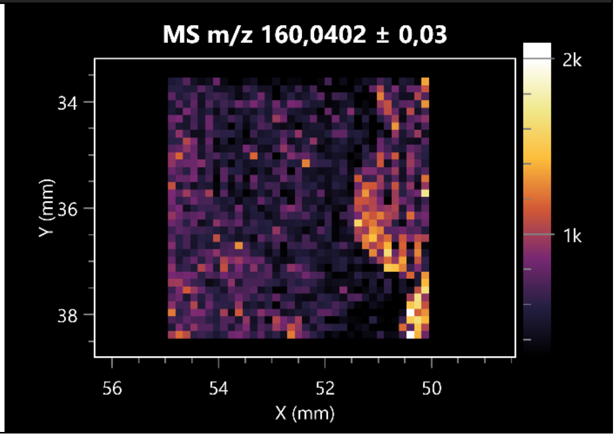

**Amines**	Aniline<br><br>(left: *B. cereus*<br>right: *F. graminarum*)	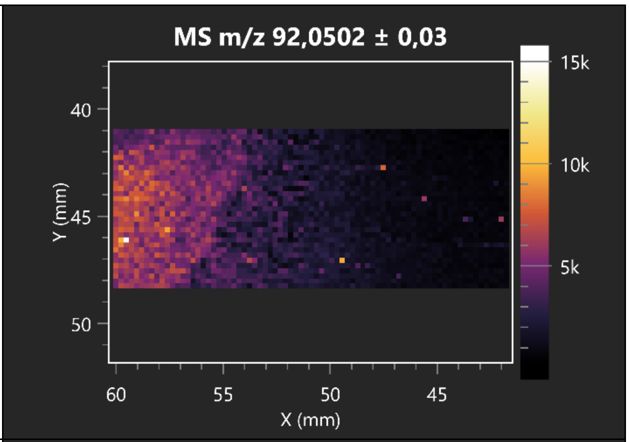
	2-Isopropyl-*N*-methylaniline<br><br>(left: *B. cereus*<br>right: *F. graminarum*)	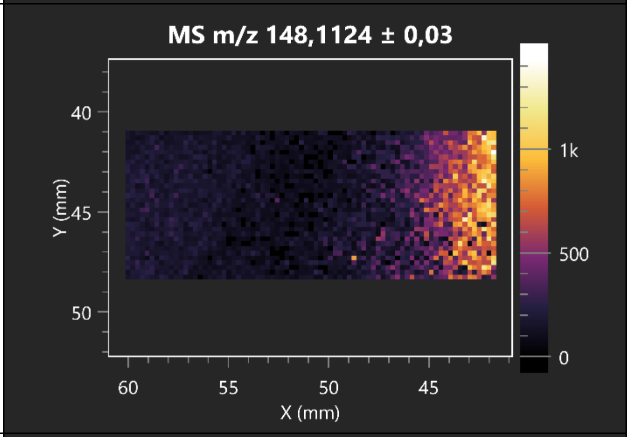
	3-Methoxytyramine<br><br>(left: *B. cereus*<br>right: *F. graminarum*)	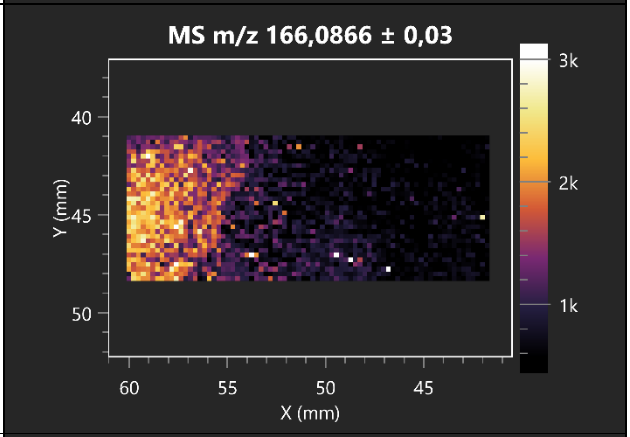
**Aldehydes**	Benzaldehyde<br><br>(left: *B. cereus*<br>right: *F. graminarum*)	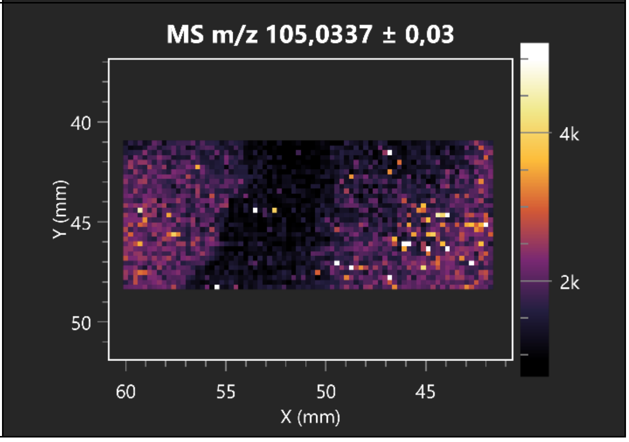

<table>
<tr>
<td rowspan="2">Purines</td>
<td>6-Dimethylaminopurine

(left: B. cereus
right: F. graminarum)</td>
<td>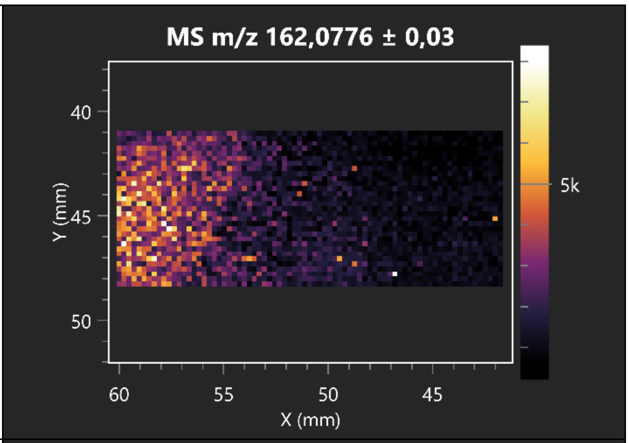
</td>
</tr>
<tr>
<td>2-Amino-6-hydroxyaminopurine

(left: B. cereus
right: F. graminarum)</td>
<td>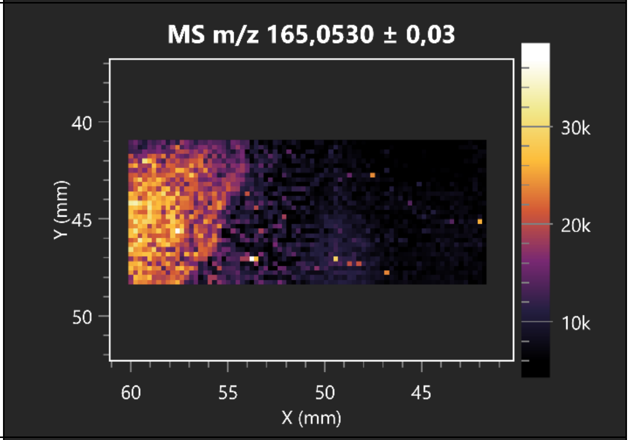
</td>
</tr>
<tr>
<td rowspan="2">Pyrimidines</td>
<td>Dihydrothymine

(left: B. cereus
right: F. graminarum)</td>
<td>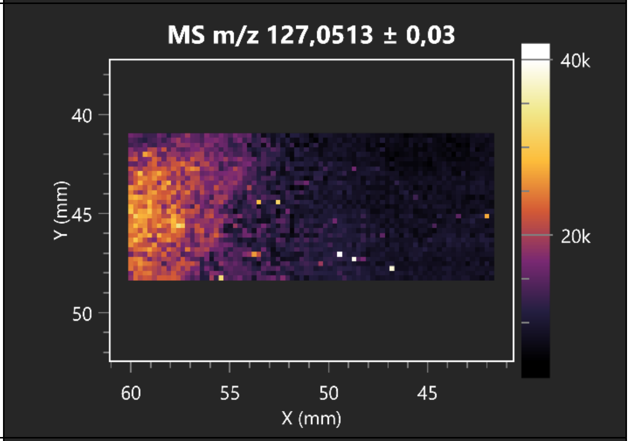
</td>
</tr>
<tr>
<td>Cytisine

(left: B. cereus
right: F. graminarum)</td>
<td>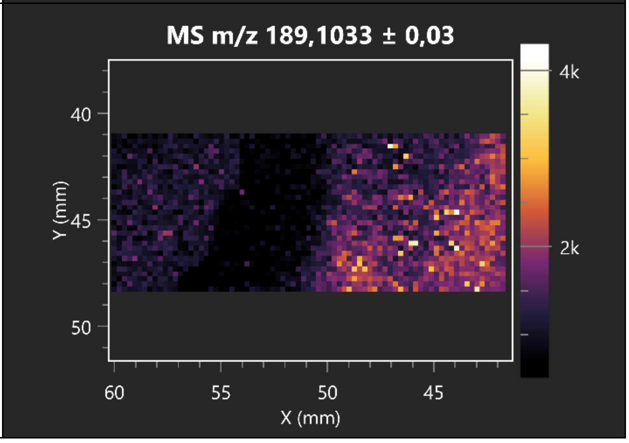
</td>
</tr>
</table>

**Benzene derivatives**	p-Octopamine<br><br>(left: *B. cereus*<br>right: *F. graminarum*)	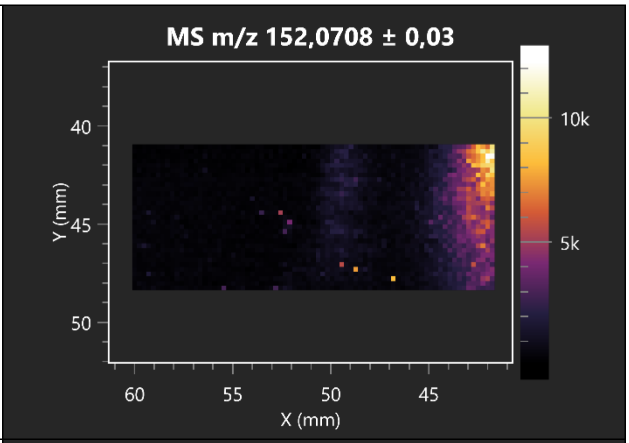
	Benzylguanidine<br><br>(left: *B. cereus*<br>right: *F. graminarum*)	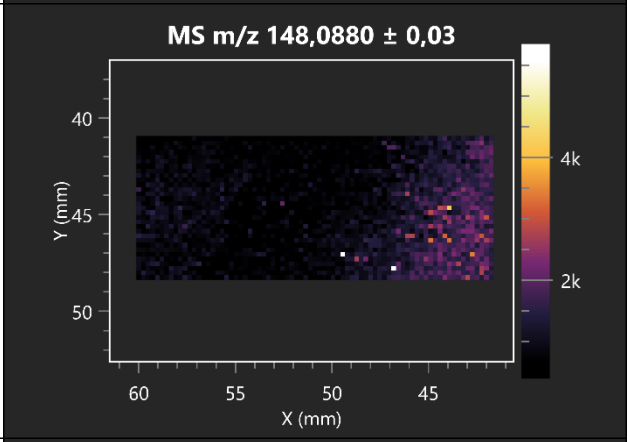
	Coumarin<br><br>(left: *B. cereus*<br>right: *F. graminarum*)	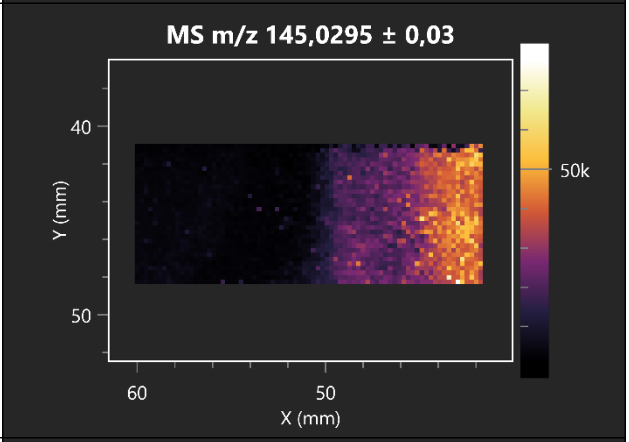
	3-Isopropylcatechol<br><br>(left: *B. cereus*<br>right: *F. graminarum*)	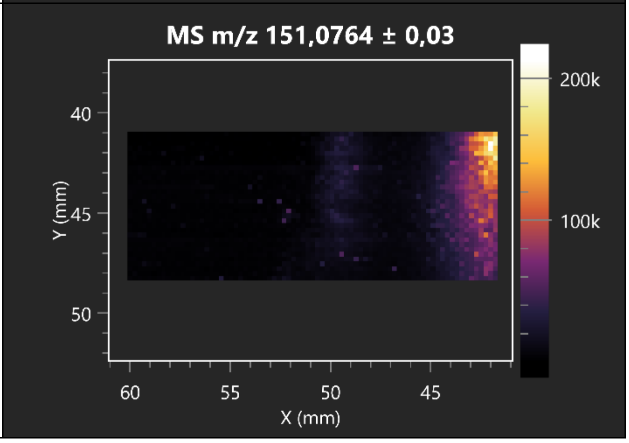

<table>
<tr><td rowspan="4">Organic phosphates</td><td>N-Methylbenzamide

(left: F. graminarum right: P. xylanexedens)</td><td>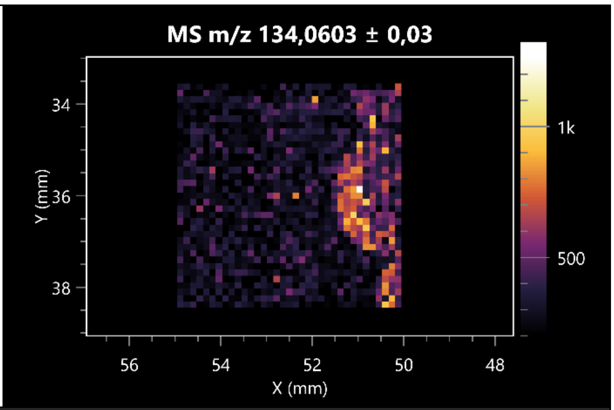
</td></tr>
<tr><td>Glycerophosphocholine

(left: B. cereus
right: F. graminarum)</td><td>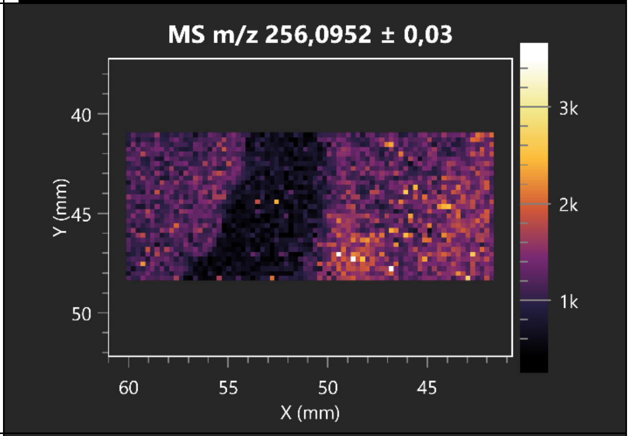
</td></tr>
<tr><td>Phosphorylcholine

(left: B. cereus
right: F. graminarum)</td><td>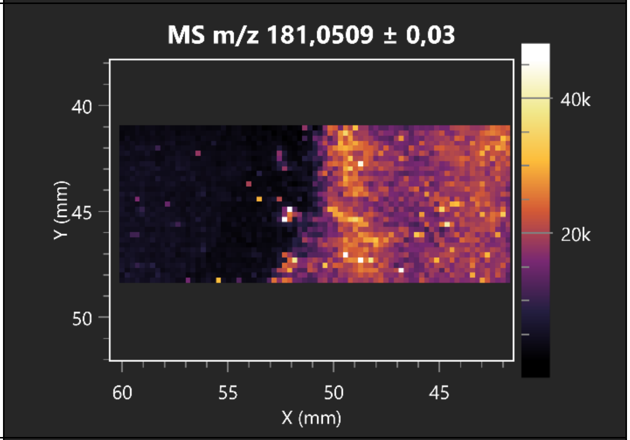
</td></tr>
<tr><td>Flavin mononucleotide

(left: B. cereus
right: F. graminarum)</td><td>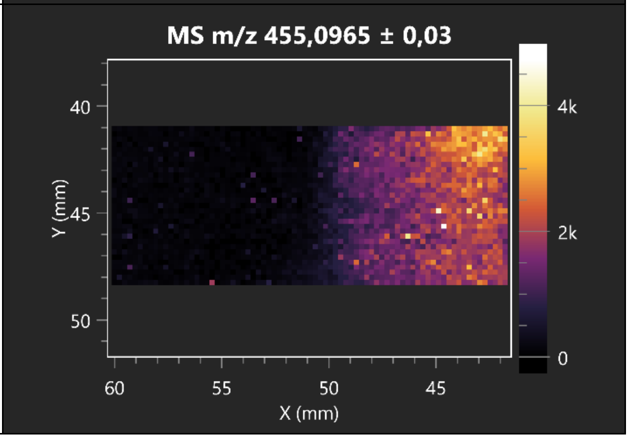
</td></tr>
</table>

**Carnitines**	Deoxycarnitine<br><br>(left: *B. cereus*<br>right: *F. graminarum*)	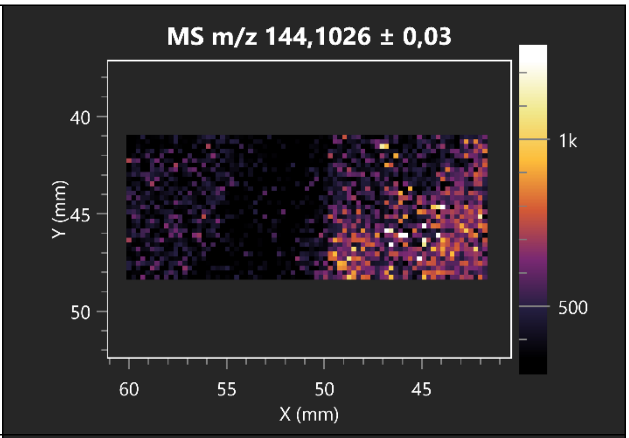
	Carnitine<br><br>(left: *B. cereus*<br>right: *F. graminarum*)	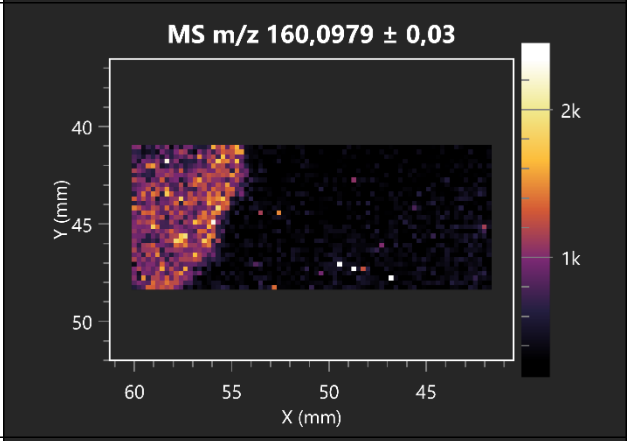
	Acetylcarnitine<br><br>(left: *B. cereus*<br>right: *F. graminarum*)	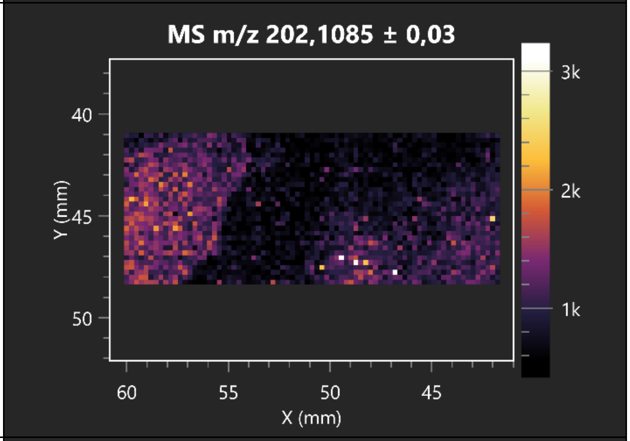
**Sulfur compounds**	Ethyl thioacetate<br><br>(left: *B. cereus*<br>right: *F. graminarum*)	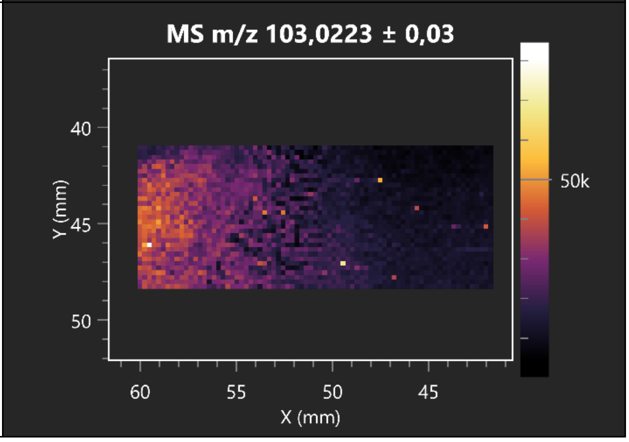

<table>
<tr><td></td><td>4-Methylbenzenesulfonic acid

(left: *B. cereus*
right: *F. graminarum*)</td><td>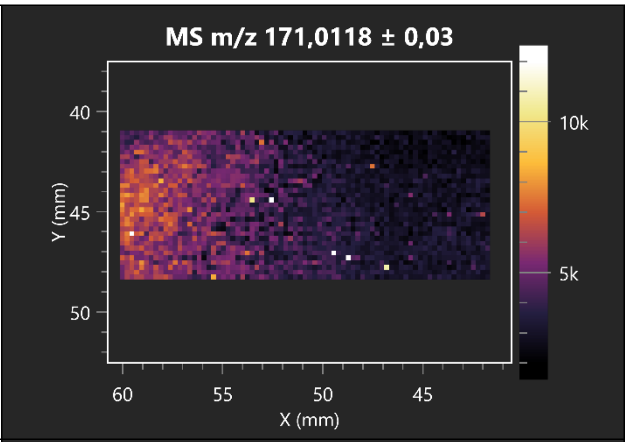
</td></tr>
<tr><td></td><td>*N*-[4-(Aminosulfonyl)phenyl]propanamide

(left: *B. cereus*
right: *F. graminarum*)</td><td>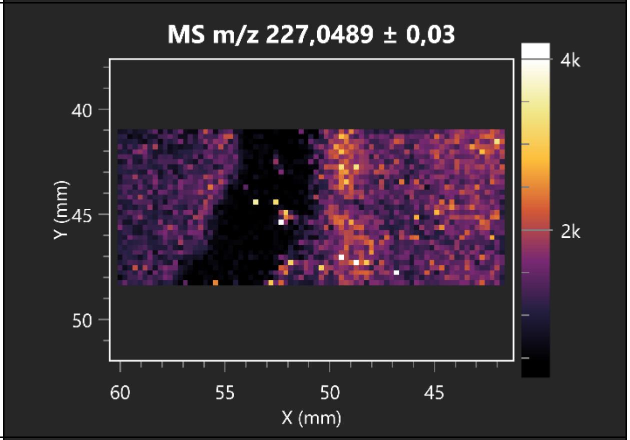
</td></tr>
<tr><td></td><td>L-Cystathionine

(left: *B. cereus*
right: *F. graminarum*)</td><td>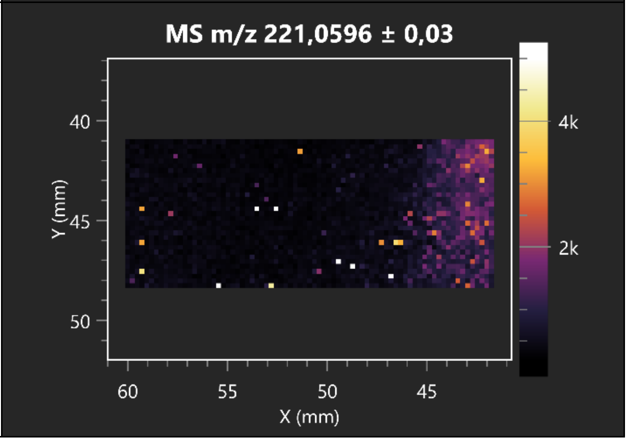
</td></tr>
</table>
