# Supplementary material for: Direct 3D Mass Spectrometry Imaging Analysis of Environmental Microorganisms
Source: Molecules. 2025 Mar 14;30(6):1317. doi: 10.3390/molecules30061317 (PMC11946574; doi:10.3390/molecules30061317)
Supplement: Supplementary file 1 [file molecules-30-01317-s001.zip › Table S2_.pdf]

Table S.2. LARAPPI/CI-MSI 3D ion images of bacterial and fungal metabolites from *Paenibacillus xylanexedens* (right) and *Fusarium graminarum* (left) culture

**Compound**		Ion images
**Amino acids and derivatives**	D-α - Aminobutyric acid	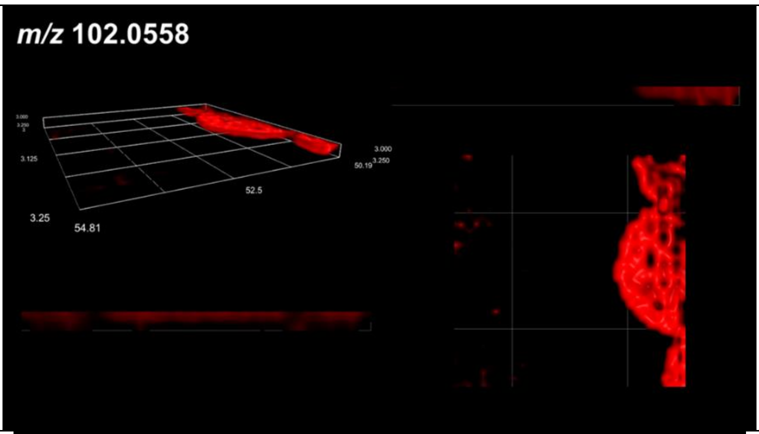
	N-Acetyl-L-alanine	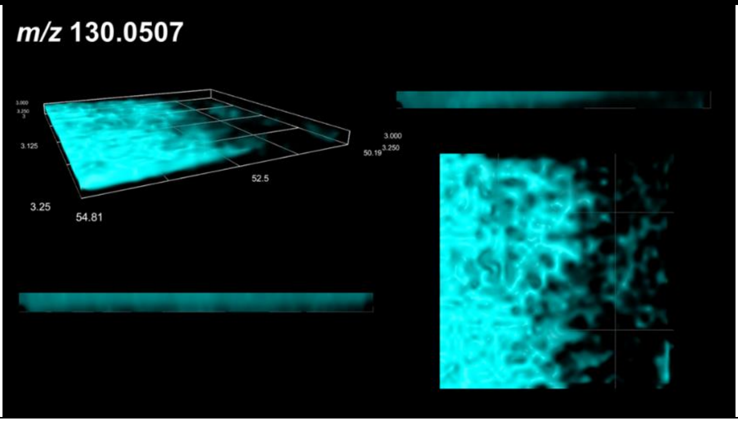
