# Supplementary material for: Direct 3D Mass Spectrometry Imaging Analysis of Environmental Microorganisms
Source: Molecules. 2025 Mar 14;30(6):1317. doi: 10.3390/molecules30061317 (PMC11946574; doi:10.3390/molecules30061317)
Supplement: Supplementary file 1 [file molecules-30-01317-s001.zip › Table S3_.pdf]

Table S3. Pathway enrichment analysis of metabolites in *Paenibacillus xylanexedens*, highlighting the matched pathways, key metabolites, and statistical significance for each metabolic pathway.

No	Pathway Name	Match Status	P-value	-log(p)	Holm p	FDR	Impact	Metabolites
1	D-Amino acid metabolism	9/29	0.0005	3.2868	0.0408	0.0408	0.5556	D-Alanine; L-Glutamate; L-Serine; D-Serine; L-Lysine; D-Lysine; D-Arginine; L-Ornithine; *N*-Acetyl-L-glutamate;
2	Arginine biosynthesis	6/18	0.0032	2.4950	0.2495	0.1264	0.4126	L-Glutamate; *N*-Acetyl-L-glutamate; L-Aspartate; L-Citrulline; L-Ornithine; 2-Oxoglutarate;
3	Arginine and proline metabolism	6/24	0.0150	1.8247	1.0000	0.3118	0.3886	Agmatine; L-Glutamate; L-Proline; L-Ornithine; Spermidine; 4-Acetamidobutanoate;
4	Glycine, serine and threonine metabolism	7/32	0.0183	1.7386	1.0000	0.3118	0.2647	L-Aspartate; L-Threonine; L-Serine; D-Serine; Tetrahydrofolate; Betaine; L-Tryptophan
5	Purine metabolism	12/72	0.0197	1.7048	1.0000	0.3118	0.2784	AMP; IMP; Xanthosine; Adenine; GMP; Hypoxanthine; Inosine; Xanthine; Deoxyguanosine; dGMP; Guanosine; 3'-AMP;
6	Cyanoamino acid metabolism	3/8	0.0273	1.5643	1.0000	0.3397	0.0000	L-Asparagine; L-Aspartate; L-Serine
7	Tyrosine metabolism	3/9	0.0383	1.4166	1.0000	0.3397	0.0000	3,4-Dihydroxyphenylethyleneglycol; L-Tyrosine; Succinate semialdehyde;
8	Alanine, aspartate and glutamate metabolism	5/22	0.0385	1.4142	1.0000	0.3397	0.6343	L-Aspartate; L-Asparagine; Succinate semialdehyde; 2-Oxoglutarate; L-Glutamate;
9	Pyrimidine metabolism	7/37	0.0387	1.4123	1.0000	0.3397	0.3390	UMP; Uracil; Uridine; Cytidine; CMP; Cytosine; Thymidine;
10	Pyruvate metabolism	5/25	0.0629	1.2011	1.0000	0.4421	0.2048	Phosphoenolpyruvate; Methyl glyoxal; (S)-Malate; Acetate; Acetaldehyde;
11	Histidine metabolism	4/18	0.0676	1.1701	1.0000	0.4421	0.1539	Urocanate; L-Histidine; L-Glutamate; Imidazole-4-acetate;
12	Glutathione metabolism	4/18	0.0676	1.1701	1.0000	0.4421	0.4142	Glutathione; Pidolic acid; L-Glutamate; Spermidine;
13	Glyoxylate and dicarboxylate metabolism	6/34	0.0727	1.1382	1.0000	0.4421	0.1818	Oxalate; Citrate; (S)-Malate; L-Glutamate; Acetate; L-Serine;
14	Taurine and hypotaurine metabolism	3/12	0.0827	1.0827	1.0000	0.4664	0.0000	Acetate; L-Glutamate; 2-Oxoglutarate
15	Citrate cycle (TCA cycle)	4/20	0.0935	1.0294	1.0000	0.4922	0.1754	2-Oxoglutarate; (S)-Malate; Citrate; Phosphoenolpyruvate

16	Methane metabolism	5/29	0.1064	0.9731	1.0000	0.5253	0.2241	Acetate; Phosphoenolpyruvate; L-Serine; (S)-Malate; Tetrahydrofolate;
17	Phenylalanine, tyrosine and tryptophan biosynthesis	4/22	0.1234	0.9088	1.0000	0.5414	0.0199	Phosphoenolpyruvate; L-Tyrosine; L-Tryptophan; L-Phenylalanine;
18	Valine, leucine and isoleucine biosynthesis	4/22	0.1234	0.9088	1.0000	0.5414	0.0882	L-Threonine; L-Leucine; 3-Methyl-2-oxobutanoic acid; 4-Methyl-2-oxopentanoate;
19	Sulfur metabolism	3/15	0.1412	0.8502	1.0000	0.5870	0.2800	L-Serine; Sulfite; Acetate
20	beta-Alanine metabolism	2/8	0.1536	0.8135	1.0000	0.6068	0.0000	L-Aspartate; Pantothenate
21	Carbapenem biosynthesis	1/3	0.2436	0.6133	1.0000	0.8748	0.0000	L-Glutamate;
22	Novobiocin biosynthesis	1/3	0.2436	0.6133	1.0000	0.8748	0.0000	L-Tyrosine;
23	Glycolysis / Gluconeogenesis	4/30	0.2726	0.5645	1.0000	0.9099	0.1188	Acetaldehyde; Phosphoenolpyruvate; Salicin; Acetate;
24	Tryptophan metabolism	2/12	0.2891	0.5390	1.0000	0.9099	0.0000	Indole-3-acetate; 5-Hydroxyindoleacetate
25	Vitamin B6 metabolism	2/12	0.2891	0.5390	1.0000	0.9099	0.0000	Pyridoxine; Pyridoxamine;
26	Pantothenate and CoA biosynthesis	3/22	0.3094	0.5095	1.0000	0.9099	0.2151	Pantothenate; 3-Methyl-2-oxobutanoic acid; L-Aspartate;
27	Biosynthesis of various plant secondary metabolites	1/4	0.3110	0.5073	1.0000	0.9099	0.0000	L-Methionine;
28	Cysteine and methionine metabolism	5/44	0.3509	0.4548	1.0000	0.9345	0.1550	5'-Methylthioadenosine; L-Methionine S-oxide; L-Methionine; L-Serine; L-Aspartate;
29	Phenylalanine metabolism	1/5	0.3724	0.4290	1.0000	0.9345	0.0000	L-Phenylalanine;
30	Biosynthesis of siderophore group nonribosomal peptides	1/5	0.3724	0.4290	1.0000	0.9345	0.3000	2,3-Dihydroxybenzoate;
31	Lysine biosynthesis	2/15	0.3904	0.4085	1.0000	0.9345	0.0000	L-Aspartate; L-Lysine
32	Nicotinate and nicotinamide metabolism	2/15	0.3904	0.4085	1.0000	0.9345	0.0000	4-Methylcatechol;
33	Lysine degradation	2/15	0.3904	0.4085	1.0000	0.9345	0.4444	L-Lysine; D-Lysine;
34	Xylene degradation	1/6	0.4284	0.3682	1.0000	0.9828	0.0000	4-Methylcatechol;
35	Propanoate metabolism	3/27	0.4354	0.3611	1.0000	0.9828	0.0075	2-Methylcitrate; Propanoate; Methylglyoxal
36	Nitrogen metabolism	1/7	0.4794	0.3193	1.0000	1.0000	0.0000	L-Glutamate;
37	Pentose and glucuronate interconversions	3/29	0.4838	0.3153	1.0000	1.0000	0.0000	L-Arabinose; D-Ribulose; 2-Oxoglutarate
38	Monobactam biosynthesis	1/8	0.5260	0.2790	1.0000	1.0000	0.0000	L-Aspartate;

39	One carbon pool by folate	1/8	0.5260	0.2790	1.0000	1.0000	0.5175	Tetrahydrofolate
40	Valine, leucine and isoleucine degradation	3/32	0.5525	0.2576	1.0000	1.0000	0.0703	L-Leucine; 3-Methyl-2-oxobutanoic acid; 4-Methyl-2-oxopentanoate;
41	Amino sugar and nucleotide sugar metabolism	3/33	0.5743	0.2409	1.0000	1.0000	0.0190	*N*-Acetyl-D-glucosamine; D-Glucosamine; L-Arabinose;
42	Butanoate metabolism	2/22	0.5963	0.2246	1.0000	1.0000	0.0171	(R)-3-Hydroxybutanoate; Succinate semialdehyde;
43	Starch and sucrose metabolism	2/27	0.7091	0.1493	1.0000	1.0000	0.0849	Maltose; alpha,alpha-Trehalose;
44	Lipoic acid metabolism	2/28	0.7283	0.1377	1.0000	1.0000	0.0247	2-Oxoglutarate; Tetrahydrofolate;
45	Riboflavin metabolism	1/16	0.7768	0.1097	1.0000	1.0000	0.1327	Riboflavin;
46	Folate biosynthesis	2/34	0.8223	0.0850	1.0000	1.0000	0.0356	Tetrahydrofolate; 4-Aminobenzoate;
47	Peptidoglycan biosynthesis	1/19	0.8320	0.0799	1.0000	1.0000	0.0000	D-Alanine;
48	Biotin metabolism	1/21	0.8610	0.0650	1.0000	1.0000	0.0792	Biotin;
49	Glycerolipid metabolism	1/21	0.8610	0.0650	1.0000	1.0000	0.1762	Phosphatidate;
50	Glycerophospholipid metabolism	1/24	0.8956	0.0479	1.0000	1.0000	0.1454	Phosphatidate;
51	Pentose phosphate pathway	1/26	0.9137	0.0392	1.0000	1.0000	0.0000	D-Glucono-1,5-lactone;
52	Galactose metabolism	1/27	0.9216	0.0355	1.0000	1.0000	0.0428	Raffinose;
53	Porphyrin metabolism	1/28	0.9287	0.0321	1.0000	1.0000	0.0779	Porphobilinogen;
54	Teichoic acid biosynthesis	1/34	0.9600	0.0177	1.0000	1.0000	0.1534	D-Alanine;
