# Supplementary material for: Direct 3D Mass Spectrometry Imaging Analysis of Environmental Microorganisms
Source: Molecules. 2025 Mar 14;30(6):1317. doi: 10.3390/molecules30061317 (PMC11946574; doi:10.3390/molecules30061317)
Supplement: Supplementary file 1 [file molecules-30-01317-s001.zip › Table S4_.pdf]

Table S4. Pathway enrichment analysis of metabolites in *Bacillus cereus*, highlighting the matched pathways, key metabolites, and statistical significance for each metabolic pathway.

No	Pathway Name	Match Status	P-value	-log(p)	Holm p	FDR	Impact	Metabolites
1	Tyrosine metabolism	3/9	0.0168	1.7743	1.0000	0.6474	0.0000	3,4-Dihydroxyphenylethyleneglycol; Succinate semialdehyde; Succinate
2	Taurine and hypotaurine metabolism	3/12	0.0382	1.4175	1.0000	0.6474	0.1429	Acetate; L-Glutamate; Acetyl phosphate;
3	Alanine, aspartate and glutamate metabolism	4/22	0.0497	1.3039	1.0000	0.6474	0.5299	L-Aspartate; Succinate semialdehyde; L-Glutamate; Succinate;
4	Glycine, serine and threonine metabolism	5/32	0.0517	1.2862	1.0000	0.6474	0.1028	L-Aspartate; O-Phospho-L-serine; Tetrahydrofolate; Betaine; L-Tryptophan
5	Nicotinate and nicotinamide metabolism	3/15	0.0688	1.1627	1.0000	0.6474	0.0000	L-Aspartate; Succinate semialdehyde; Succinate
6	Sulfur metabolism	3/15	0.0688	1.1627	1.0000	0.6474	0.3126	Sulfite; Acetate; Succinate
7	Pyruvate metabolism	4/25	0.0742	1.1297	1.0000	0.6474	0.3333	Methylglyoxal; Acetate; Acetaldehyde; Acetyl phosphate;
8	beta-Alanine metabolism	2/8	0.0911	1.0405	1.0000	0.6474	0.0000	L-Aspartate; Pantothenate
9	Propanoate metabolism	4/27	0.0934	1.0297	1.0000	0.6474	0.0101	2-Methylcitrate; Succinate; 2-Hydroxybutanoic acid; Methylglyoxal
10	Galactose metabolism	4/27	0.0934	1.0297	1.0000	0.6474	0.0629	Raffinose; D-Fructose; D-Glucose; D-Sorbitol;
11	Starch and sucrose metabolism	4/27	0.0934	1.0297	1.0000	0.6474	0.2595	D-Fructose; D-Glucose; Maltose; alpha,alpha-Trehalose;
12	Histidine metabolism	3/18	0.1074	0.9692	1.0000	0.6474	0.0846	L-Histidine; L-Glutamate; Imidazole-4-acetate;
13	Arginine biosynthesis	3/18	0.1074	0.9692	1.0000	0.6474	0.1719	L-Glutamate; L-Aspartate; L-Citrulline;
14	Methane metabolism	4/29	0.1147	0.9403	1.0000	0.6474	0.1224	O-Phospho-L-serine; Acetate; Acetyl phosphate; Tetrahydrofolate;
15	Glycolysis / Gluconeogenesis	4/30	0.1262	0.8991	1.0000	0.6592	0.0328	Acetaldehyde; D-Glucose; Salicin; Acetate;
16	Fructose and mannose metabolism	3/20	0.1369	0.8638	1.0000	0.6592	0.2060	D-Fructose; D-Sorbitol; Mannitol;
17	Cysteine and methionine metabolism	5/44	0.1532	0.8147	1.0000	0.6592	0.1673	5'-Methylthioadenosine; L-Methionine S-oxide; L-Methionine; L-Aspartate; O-Phospho-L-serine;
18	Amino sugar and nucleotide sugar metabolism	4/33	0.1631	0.7875	1.0000	0.6592	0.0000	D-Glucosamine; D-Glucose; D-Fructose; L-Arabinose;
19	Butanoate metabolism	3/22	0.1688	0.7727	1.0000	0.6592	0.0342	(R)-3-Hydroxybutanoate; Succina

								te semialdehyde; Succinate;
20	Glyoxylate and dicarboxylate metabolism	4/34	0.1763	0.7539	1.0000	0.6592	0.1818	Oxalate; Citrate; L-Glutamate; Acetate;
21	Vitamin B6 metabolism	2/12	0.1819	0.7402	1.0000	0.6592	0.0000	Pyridoxine; Pyridoxamine;
22	Carbapenem biosynthesis	1/3	0.1836	0.7362	1.0000	0.6592	0.0000	L-Glutamate;
23	Arginine and proline metabolism	3/24	0.2027	0.6932	1.0000	0.6961	0.0000	L-Glutamate; L-Proline; *N*-Acetylputrescine;
24	Biosynthesis of various plant secondary metabolites	1/4	0.2371	0.6251	1.0000	0.7781	0.0000	L-Methionine;
25	Lysine biosynthesis	2/15	0.2561	0.5916	1.0000	0.7781	0.0000	L-Aspartate; L-Lysine
26	Lysine degradation	2/15	0.2561	0.5916	1.0000	0.7781	0.2222	L-Lysine; Glutarate;
27	Phenylalanine metabolism	1/5	0.2871	0.5420	1.0000	0.8100	0.0000	L-Phenylalanine;
28	Biosynthesis of siderophore group nonribosomal peptides	1/5	0.2871	0.5420	1.0000	0.8100	0.3000	2,3-Dihydroxybenzoate
29	Glutathione metabolism	2/18	0.3310	0.4802	1.0000	0.8655	0.1716	Pidolic acid; L-Glutamate;
30	Xylene degradation	1/6	0.3339	0.4764	1.0000	0.8655	0.0000	4-Methylcatechol;
31	Valine, leucine and isoleucine degradation	3/32	0.3484	0.4579	1.0000	0.8655	0.0352	L-Isoleucine; 4-Methyl-2-oxopentanoate; Methylmalonate;
32	Nitrogen metabolism	1/7	0.3777	0.4229	1.0000	0.8655	0.0000	L-Glutamate;
33	Citrate cycle (TCA cycle)	2/20	0.3799	0.4204	1.0000	0.8655	0.1191	Succinate; Citrate;
34	Monobactam biosynthesis	1/8	0.4186	0.3782	1.0000	0.8655	0.0000	L-Aspartate;
35	Cyanoamino acid metabolism	1/8	0.4186	0.3782	1.0000	0.8655	0.0000	L-Aspartate;
36	One carbon pool by folate	1/8	0.4186	0.3782	1.0000	0.8655	0.5175	Tetrahydrofolate
37	Valine, leucine and isoleucine biosynthesis	2/22	0.4273	0.3693	1.0000	0.8655	0.0000	L-Isoleucine; 4-Methyl-2-oxopentanoate;
38	Phenylalanine, tyrosine and tryptophan biosynthesis	2/22	0.4273	0.3693	1.0000	0.8655	0.0199	L-Tryptophan;
39	Pantothenate and CoA biosynthesis	2/22	0.4273	0.3693	1.0000	0.8655	0.1395	L-Phenylalanine;
40	Pyrimidine metabolism	3/37	0.4400	0.3565	1.0000	0.8690	0.1909	Uracil; Uridine; Cytidine;
41	Streptomycin biosynthesis	1/10	0.4927	0.3074	1.0000	0.9484	0.0000	D-Glucose
42	Purine metabolism	5/72	0.5148	0.2884	1.0000	0.9484	0.0582	Xanthosine; Hypoxanthine; Inosine; dGMP; Guanosine;
43	Pentose phosphate pathway	2/26	0.5162	0.2872	1.0000	0.9484	0.0376	D-Gluconic acid; D-Glucono-1,5-lactone;
44	Tryptophan metabolism	1/12	0.5574	0.2538	1.0000	1.0000	0.0000	5-Hydroxyindoleacetate
45	D-Amino acid metabolism	2/29	0.5768	0.2390	1.0000	1.0000	0.0889	L-Glutamate; L-Lysine;
46	Riboflavin metabolism	1/16	0.6635	0.1781	1.0000	1.0000	0.1327	Riboflavin;

47	Folate biosynthesis	2/34	0.6654	0.1770	1.0000	1.0000	0.0356	Tetrahydrofolate; 4-Aminobenzoate;
48	Biotin metabolism	1/21	0.7615	0.1183	1.0000	1.0000	0.0792	Biotin;
49	Glycerolipid metabolism	1/21	0.7615	0.1183	1.0000	1.0000	0.1762	Phosphatidate;
50	Glycerophospholipid metabolism	1/24	0.8062	0.0936	1.0000	1.0000	0.1454	Phosphatidate;
51	Lipoic acid metabolism	1/28	0.8532	0.0690	1.0000	1.0000	0.0247	Tetrahydrofolate;
52	Pentose and glucuronate interconversions	1/29	0.8630	0.0640	1.0000	1.0000	0.0000	L-Arabinose;
