# Supplementary material for: Direct 3D Mass Spectrometry Imaging Analysis of Environmental Microorganisms
Source: Molecules. 2025 Mar 14;30(6):1317. doi: 10.3390/molecules30061317 (PMC11946574; doi:10.3390/molecules30061317)
Supplement: Supplementary file 1 [file molecules-30-01317-s001.zip › Table S5_.pdf]

Table S5. Pathway enrichment analysis of metabolites in *Fusarium graminarum*, highlighting the matched pathways, key metabolites, and statistical significance for each metabolic pathway.

No	Pathway Name	Match Status	P-value	-log(p)	Holm p	FDR	Impact	Metabolites
1	Alanine, aspartate and glutamate metabolism	7/22	0.0020	2.7063	0.1691	0.1532	0.8058	L-Aspartate; N6-(1,2-Dicarboxyethyl)-AMP; Succinate semialdehyde; L-Glutamine; 2-Oxoglutarate; L-Glutamate; Succinate;
2	Arginine biosynthesis	6/19	0.0044	2.3571	0.3735	0.1532	0.2504	L-Glutamate; *N*-Acetyl-L-glutamate; L-Aspartate; L-Citrulline; 2-Oxoglutarate; L-Glutamine
3	Purine metabolism	13/72	0.0080	2.0968	0.6722	0.1532	0.3151	L-Glutamine; AMP; N6-(1,2-Dicarboxyethyl)-AMP; IMP; Xanthosine; Adenine; Hypoxanthine; Inosine; GMP; Deoxyguanosine; Urate; dGMP; Guanosine;
4	Butanoate metabolism	5/16	0.0099	2.0061	0.8184	0.1532	0.1333	L-Glutamate; Succinate semialdehyde; 2-Oxoglutarate; Succinate; Acetone
5	Lysine biosynthesis	5/16	0.0099	2.0061	0.8184	0.1532	0.1860	L-Aspartate; N6-(L-1,3-Dicarboxypropyl)-L-lysine; L-2-Aminoadipate; 2-Oxoglutarate; L-Lysine;
6	Glyoxylate and dicarboxylate metabolism	7/29	0.0107	1.9711	0.8658	0.1532	0.0752	Oxalate; Citrate; (S)-Malate; L-Glutamate; Acetate; Succinate; L-Glutamine
7	Pyruvate metabolism	6/24	0.0151	1.8208	1.0000	0.1856	0.2803	Phosphoenolpyruvate; (S)-Malate; Acetate; Acetaldehyde; Methylglyoxal; alpha-Isopropylmalate;
8	Citrate cycle (TCA cycle)	5/20	0.0263	1.5803	1.0000	0.2826	0.2258	2-Oxoglutarate; Succinate; (S)-Malate; Citrate; Phosphoenolpyruvate
9	Glycine, serine and threonine metabolism	7/37	0.0390	1.4085	1.0000	0.3731	0.2011	L-Aspartate; O-Phospho-L-serine; L-Threonine; Tetrahydrofolate; Betaine; Methylglyoxal; L-Tryptophan;
10	Phenylalanine, tyrosine and tryptophan biosynthesis	5/23	0.0461	1.3363	1.0000	0.3965	0.1144	Shikimate; Quinate; Phosphoenolpyruvate; L-Tryptophan; L-Phenylalanine;
11	Taurine and hypotaurine metabolism	3/10	0.0514	1.2891	1.0000	0.4018	0.0000	Acetate; L-Glutamate; 2-Oxoglutarate
12	Pyrimidine metabolism	7/41	0.0636	1.1967	1.0000	0.4281	0.2477	L-Glutamine; UMP; Uracil; Uridine; CMP; Cytidine; Thymidine;
13	Amino sugar and nucleotide sugar metabolism	6/33	0.0647	1.1890	1.0000	0.4281	0.2575	*N*-Acetyl-D-glucosamine 6-phosphate; D-Mannose 6-phosphate; UDP-*N*-acetyl-alpha-D-glucosamine; UDP-alpha-D-

								galactose;; *N*-Acetyl-D-glucosamine; L-Arabinose;
14	Nicotinate and nicotinamide metabolism	3/12	0.0828	1.0821	1.0000	0.4746	0.0197	Succinate semialdehyde; Nicotinamide; Succinate
15	Vitamin B6 metabolism	3/12	0.0828	1.0821	1.0000	0.4746	0.0476	L-Glutamine; Pyridoxine; Pyridoxamine;
16	Valine, leucine and isoleucine biosynthesis	4/20	0.0937	1.0284	1.0000	0.5035	0.1675	L-Threonine; alpha-Isopropylmalate; 3-Methyl-2-oxobutanoic acid; 4-Methyl-2-oxopentanoate;
17	Tyrosine metabolism	5/32	0.1469	0.8329	1.0000	0.7433	0.0740	3,4-Dihydroxyphenylethyleneglycol; Dopamine; Succinate semialdehyde; Homovanillate; Succinate;
18	Methane metabolism	4/25	0.1749	0.7572	1.0000	0.7916	0.2344	O-Phospho-L-serine; Acetate; Phosphoenolpyruvate; Tetrahydrofolate;
19	Glutathione metabolism	4/25	0.1749	0.7572	1.0000	0.7916	0.4744	Glutathione disulfide; L-Glutamate; Glutathione; Pidolic acid;
20	Histidine metabolism	3/19	0.2341	0.6306	1.0000	0.9521	0.0788	L-Histidine; Imidazole-4-acetate; Ergothioneine
21	Carbapenem biosynthesis	1/3	0.2436	0.6134	1.0000	0.9521	0.0000	L-Glutamate;
22	Indole alkaloid biosynthesis	1/3	0.2436	0.6134	1.0000	0.9521	1.0000	L-Tryptophan;
23	Nitrogen metabolism	2/12	0.2891	0.5390	1.0000	1.0000	0.0000	L-Glutamine; L-Glutamate;
24	Riboflavin metabolism	2/12	0.2891	0.5390	1.0000	1.0000	0.4333	Riboflavin; FMN;
25	Arginine and proline metabolism	4/31	0.2934	0.5325	1.0000	1.0000	0.0234	Agmatine; L-Proline; L-Glutamate; 4-Acetamidobutanoate;
26	Monobactam biosynthesis	1/4	0.3109	0.5074	1.0000	1.0000	0.0000	L-Aspartate
27	Lysine degradation	3/23	0.3348	0.4752	1.0000	1.0000	0.0400	L-Lysine; N6-(L-1,3-Dicarboxypropyl)-L-lysine; L-Pipecolate;
28	Sulfur metabolism	2/14	0.3571	0.4473	1.0000	1.0000	0.1000	Acetate; Succinate;
29	Propanoate metabolism	3/24	0.3602	0.4435	1.0000	1.0000	0.0324	Propanoate; Succinate; 2-Hydroxybutanoic acid;
30	Linoleic acid metabolism	1/5	0.3723	0.4291	1.0000	1.0000	0.6250	Linoleate;
31	Glycerophospholipid metabolism	4/35	0.3777	0.4228	1.0000	1.0000	0.2632	1-Acyl-s*N*-glycero-3-phosphocholine; Choline; s*N*-Glycerol 3-phosphate; s*N*-Glycero-3-phosphocholine;
32	Glycolysis / Gluconeogenesis	3/25	0.3855	0.4140	1.0000	1.0000	0.1409	Acetaldehyde; Phosphoenolpyruvate; Acetate;
33	Fructose and mannose metabolism	3/25	0.3855	0.4140	1.0000	1.0000	0.1947	Mannitol; D-Mannose 6-phosphate; L-Sorbose;
34	beta-Alanine metabolism	2/16	0.4227	0.3740	1.0000	1.0000	0.0000	L-Aspartate; Pantothenate;

35	Biosynthesis of various plant secondary metabolites	1/6	0.4283	0.3683	1.0000	1.0000	0.0000	L-Methionine;
36	Biosynthesis of various other secondary metabolites	1/7	0.4793	0.3194	1.0000	1.0000	0.0000	L-Tryptophan;
37	Folate biosynthesis	3/30	0.5070	0.2950	1.0000	1.0000	0.1466	Tetrahydrofolate; 4-Aminobenzoate; Dihydrobiopterin;
38	One carbon pool by folate	1/8	0.5258	0.2792	1.0000	1.0000	0.3606	Tetrahydrofolate;
39	Cysteine and methionine metabolism	4/43	0.5409	0.2669	1.0000	1.0000	0.1422	5'-Methylthioadenosine; L-Methionine; L-Aspartate; O-Phospho-L-serine;
40	Starch and sucrose metabolism	2/22	0.5959	0.2248	1.0000	1.0000	0.0743	alpha,alpha-Trehalose; Maltose;
41	Phenylalanine metabolism	1/10	0.6069	0.2169	1.0000	1.0000	0.4839	L-Phenylalanine;
42	Porphyrin metabolism	2/23	0.6208	0.2071	1.0000	1.0000	0.0582	Porphobilinogen; L-Glutamate;
43	Pantothenate and CoA biosynthesis	2/23	0.6208	0.2071	1.0000	1.0000	0.2934	Pantothenate; 3-Methyl-2-oxobutanoic acid;
44	Tryptophan metabolism	3/37	0.6538	0.1846	1.0000	1.0000	0.0935	L-Tryptophan; 5-Hydroxy-L-tryptophan; 5-Hydroxyindoleacetate
45	Galactose metabolism	2/27	0.7086	0.1496	1.0000	1.0000	0.0369	Stachyose; UDP-alpha-D-galactose;
46	Pentose and glucuronate interconversions	2/27	0.7086	0.1496	1.0000	1.0000	0.0472	D-Arabitol; L-Arabinose;
47	Lipoic acid metabolism	2/28	0.7277	0.1380	1.0000	1.0000	0.0037	2-Oxoglutarate; Tetrahydrofolate;
48	Biotin metabolism	1/15	0.7543	0.1225	1.0000	1.0000	0.1282	Biotin;
49	Glycerolipid metabolism	1/16	0.7764	0.1099	1.0000	1.0000	0.0946	s*N*-Glycerol 3-phosphate;
50	Pentose phosphate pathway	1/18	0.8149	0.0889	1.0000	1.0000	0.0000	D-Gluconic acid;
51	Ubiquinone and other terpenoid-quinone biosynthesis	1/18	0.8149	0.0889	1.0000	1.0000	0.0000	4-Coumarate;
52	Terpenoid backbone biosynthesis	1/18	0.8149	0.0889	1.0000	1.0000	0.1147	(R)-Mevalonate;
53	Valine, leucine and isoleucine degradation	2/36	0.8459	0.0727	1.0000	1.0000	0.0215	3-Methyl-2-oxobutanoic acid; 4-Methyl-2-oxopentanoate;
54	Cyanoamino acid metabolism	1/24	0.8952	0.0481	1.0000	1.0000	0.0000	L-Aspartate;
55	Sphingolipid metabolism	1/24	0.8952	0.0481	1.0000	1.0000	0.2500	Sphinganine;
