# Supplementary material for: Direct 3D Mass Spectrometry Imaging Analysis of Environmental Microorganisms
Source: Molecules. 2025 Mar 14;30(6):1317. doi: 10.3390/molecules30061317 (PMC11946574; doi:10.3390/molecules30061317)
Supplement: Supplementary file 1 [file molecules-30-01317-s001.zip › Table S6_.pdf]

Table S6. Enrichment analysis of main-class chemical structures in *Paenibacillus xylanexedens*

No	Metabolite Set	Total	Hits	Hits [%]	Expect	P value	Holm P	FDR
1	Carboxylic acids and derivatives	3740	101	39.9	4.56	4.96E-106	2.36E-103	2.36E-103
2	Organooxygen compounds	3160	35	13.8	3.84	7.77E-23	3.69E-20	1.85E-20
3	Indoles and derivatives	559	10	4.0	0.68	2.51E-09	1.19E-06	3.99E-07
4	Keto acids and derivatives	114	6	2.4	0.139	7.31E-09	3.46E-06	8.69E-07
5	Imidazopyrimidines	198	6	2.4	0.241	1.95E-07	9.20E-05	1.86E-05
6	Purine nucleosides	121	5	2.0	0.147	4.54E-07	2.14E-04	3.60E-05
7	Purine nucleotides	134	5	2.0	0.163	7.53E-07	3.54E-04	5.12E-05
8	Phenols	434	7	2.8	0.528	1.28E-06	6.01E-04	7.62E-05
9	Pyrimidine nucleosides	87	4	1.6	0.106	4.40E-06	0.0021	2.33E-04
10	Benzene and substituted derivatives	3050	15	5.9	3.71	6.55E-06	0.0031	3.12E-04
11	Organic phosphoric acids and derivatives	93	3	1.2	0.113	2.13E-04	0.0993	0.0092
12	Pteridines and derivatives	100	3	1.2	0.122	2.64E-04	0.1230	0.0105
13	Non-metal oxoanionic compounds	28	2	0.8	0.0341	5.46E-04	0.2530	0.0200
14	Furans	30	2	0.8	0.0365	6.27E-04	0.2900	0.0206
15	Lactones	136	3	1.2	0.165	6.48E-04	0.2990	0.0206
16	Organonitrogen compounds	618	5	2.0	0.752	0.0010	0.4770	0.0308
17	Fatty Acyls	4680	14	5.5	5.7	0.0020	0.9290	0.0566
18	Pyrimidine nucleotides	77	2	0.8	0.0937	0.0041	1.0000	0.1040
19	Phenylpropanoic acids	78	2	0.8	0.0949	0.0042	1.0000	0.1040
20	Diazines	342	3	1.2	0.416	0.0087	1.0000	0.2040
21	Hydroxy acids and derivatives	116	2	0.8	0.141	0.0090	1.0000	0.2040
22	Organic sulfuric acids and derivatives	122	2	0.8	0.148	0.0099	1.0000	0.2140
23	Ribonucleoside 3'-phosphates	9	1	0.4	0.011	0.0109	1.0000	0.2260
24	Biotin and derivatives	14	1	0.4	0.017	0.0169	1.0000	0.3350
25	Azoles	462	3	1.2	0.562	0.0193	1.0000	0.3680
26	Cinnamaldehydes	18	1	0.4	0.0219	0.0217	1.0000	0.3970
27	5'-deoxyribonucleosides	27	1	0.4	0.0329	0.0323	1.0000	0.5700
28	Oxanes	35	1	0.4	0.0426	0.0417	1.0000	0.7090
29	Oxepanes	37	1	0.4	0.045	0.0441	1.0000	0.7230
30	Organic phosphonic acids and derivatives	53	1	0.4	0.0645	0.0625	1.0000	0.9920
31	Pyridines and derivatives	418	2	0.8	0.509	0.0926	1.0000	1.0000
32	Cinnamic acids and derivatives	300	1	0.4	0.365	0.3060	1.0000	1.0000
33	Prenol lipids	3830	2	0.8	4.66	0.9480	1.0000	1.0000
34	Glycerophospholipids	40000	1	0.4	48.7	1.0000	1.0000	1.0000
