# Supplementary material for: Direct 3D Mass Spectrometry Imaging Analysis of Environmental Microorganisms
Source: Molecules. 2025 Mar 14;30(6):1317. doi: 10.3390/molecules30061317 (PMC11946574; doi:10.3390/molecules30061317)
Supplement: Supplementary file 1 [file molecules-30-01317-s001.zip › Table S7_.pdf]

Table S7. Enrichment analysis of main-class chemical structures in *Bacillus cereus*

No	Metabolite Set	Total	Hits	Hits [%]	Expect	P value	Holm P	FDR
1	Carboxylic acids and derivatives	3740	76	32,2	4.25	1.06E-71	5.03E-69	5.03E-69
2	Organooxygen compounds	3160	35	14,8	3.58	7.25E-24	3.44E-21	1.73E-21
3	Indoles and derivatives	559	10	4,2	0.634	1.29E-09	6.11E-07	2.04E-07
4	Benzene and substituted derivatives	3050	19	8,0	3.46	3.23E-09	1.53E-06	3.85E-07
5	Phenols	434	8	3,4	0.493	4.72E-08	2.23E-05	4.50E-06
6	Imidazopyrimidines	198	6	2,5	0.225	1.30E-07	6.11E-05	1.03E-05
7	Fatty Acyls	4680	19	8,0	5.32	2.22E-06	0.0010	1.51E-04
8	Keto acids and derivatives	114	4	1,7	0.129	9.79E-06	0.0046	5.54E-04
9	Hydroxy acids and derivatives	116	4	1,7	0.132	1.05E-05	0.0049	5.54E-04
10	Purine nucleosides	121	4	1,7	0.137	1.24E-05	0.0058	5.89E-04
11	Phenylpropanoic acids	78	3	1,3	0.0885	1.03E-04	0.0481	0.0045
12	Pyrimidine nucleosides	87	3	1,3	0.0987	1.43E-04	0.0663	0.0057
13	Pteridines and derivatives	100	3	1,3	0.114	2.15E-04	0.0999	0.0079
14	Non-metal oxoanionic compounds	28	2	0,8	0.0318	4.76E-04	0.2200	0.0162
15	Carboximidic acids and derivatives	38	2	0,8	0.0431	8.78E-04	0.4060	0.0279
16	Organic phosphoric acids and derivatives	93	2	0,8	0.106	0.0051	1.0000	0.1530
17	Organonitrogen compounds	618	4	1,7	0.701	0.0057	1.0000	0.1590
18	Diazines	342	3	1,3	0.388	0.0072	1.0000	0.1900
19	Organic sulfuric acids and derivatives	122	2	0,8	0.138	0.0087	1.0000	0.2170
20	Pyridines and derivatives	418	3	1,3	0.474	0.0124	1.0000	0.2940
21	Biotin and derivatives	14	1	0,4	0.0159	0.0158	1.0000	0.3580
22	5'-deoxyribonucleosides	27	1	0,4	0.0306	0.0302	1.0000	0.6530
23	Furans	30	1	0,4	0.0341	0.0335	1.0000	0.6930
24	Oxanes	35	1	0,4	0.0397	0.0390	1.0000	0.7730
25	Oxepanes	37	1	0,4	0.042	0.0412	1.0000	0.7840
26	Organic carbonic acids and derivatives	63	1	0,4	0.0715	0.0691	1.0000	1.0000
27	Benzimidazoles	87	1	0,4	0.0987	0.0941	1.0000	1.0000
28	Azoles	462	2	0,8	0.524	0.0974	1.0000	1.0000
29	Linear 1,3-diarylpropanoids	106	1	0,4	0.12	0.1130	1.0000	1.0000
30	Purine nucleotides	134	1	0,4	0.152	0.1410	1.0000	1.0000
31	Lactones	136	1	0,4	0.154	0.1430	1.0000	1.0000
32	Cinnamic acids and derivatives	300	1	0,4	0.341	0.2890	1.0000	1.0000
33	Naphthalenes	334	1	0,4	0.379	0.3160	1.0000	1.0000
34	Coumarins and derivatives	341	1	0,4	0.387	0.3210	1.0000	1.0000
35	Quinolines and derivatives	416	1	0,4	0.472	0.3770	1.0000	1.0000
36	Prenol lipids	3830	5	2,1	4.34	0.4380	1.0000	1.0000
37	Steroids and steroid derivatives	2040	2	0,8	2.31	0.6740	1.0000	1.0000
38	Glycerophospholipids	40000	1	0,4	45.4	1.0000	1.0000	1.0000
