# Supplementary material for: Direct 3D Mass Spectrometry Imaging Analysis of Environmental Microorganisms
Source: Molecules. 2025 Mar 14;30(6):1317. doi: 10.3390/molecules30061317 (PMC11946574; doi:10.3390/molecules30061317)
Supplement: Supplementary file 1 [file molecules-30-01317-s001.zip › Table S8_.pdf]

Table S8. Enrichment analysis of main-class chemical structures in *Fusarium graminarum*

No	Metabolite Set	Total	Hits	Hits [%]	Expect	P-value	Holm P	FDR
1	Carboxylic acids and derivatives	3740	81	33.8	4.32	3.10E-78	1.48E-75	1.48E-75
2	Organooxygen compounds	3160	34	14.2	3.64	1.44E-22	6.85E-20	3.43E-20
3	Imidazopyrimidines	198	9	3,8	0.229	2.77E-12	1.31E-09	4.40E-10
4	Phenols	434	10	4.2	0.501	1.33E-10	6.31E-08	1.59E-08
5	Pyrimidine nucleosides	87	5	2.1	0.1	6.72E-08	3.17E-05	6.40E-06
6	Keto acids and derivatives	114	5	2.1	0.132	2.60E-07	1.23E-04	2.07E-05
7	Indoles and derivatives	559	8	3.3	0.645	3.65E-07	1.72E-04	2.48E-05
8	Pyrimidine nucleotides	77	4	1.7	0.0889	2.19E-06	0.0010	1.30E-04
9	Benzene and substituted derivatives	3050	15	6.2	3.52	3.46E-06	0.0016	1.83E-04
10	Purine nucleosides	121	4	1.7	0.14	1.32E-05	0.0062	6.30E-04
11	Purine nucleotides	134	4	1.7	0.155	1.98E-05	0.0092	8.55E-04
12	Fatty Acyls	4680	17	7.1	5.41	3.87E-05	0.0180	0.0015
13	Organic phosphoric acids and derivatives	93	3	1.2	0.107	1.83E-04	0.0847	0.0067
14	Hydroxy acids and derivatives	116	3	1.2	0.134	3.50E-04	0.1620	0.0119
15	Pyridines and derivatives	418	4	1.7	0.482	0.0015	0.6900	0.0474
16	Phenylpropanoic acids	78	2	0.8	0.09	0.0038	1.0000	0.1120
17	Organonitrogen compounds	618	4	1.7	0.713	0.0060	1.0000	0.1610
18	Pteridines and derivatives	100	2	0.8	0.115	0.0061	1.0000	0.1610
19	Flavin nucleotides	6	1	0.4	0.00693	0.0069	1.0000	0.1730
20	Organic sulfuric acids and derivatives	122	2	0.8	0.141	0.0089	1.0000	0.2130
21	Lactones	136	2	0.8	0.157	0.0110	1.0000	0.2490
22	Tropones	11	1	0.4	0.0127	0.0126	1.0000	0.2730
23	Biotin and derivatives	14	1	0.4	0.0162	0.0160	1.0000	0.3320
24	5'-deoxyribonucleosides	27	1	0.4	0.0312	0.0307	1.0000	0.6060
25	Non-metal oxoanionic compounds	28	1	0.4	0.0323	0.0318	1.0000	0.6060
26	Furans	30	1	0.4	0.0346	0.0341	1.0000	0.6240
27	Oxanes	35	1	0.4	0.0404	0.0396	1.0000	0.6870
28	Tropane alkaloids	36	1	0.4	0.0416	0.0407	1.0000	0.6870
29	Oxepanes	37	1	0.4	0.0427	0.0418	1.0000	0.6870
30	Azoles	462	2	0.8	0.533	0.1000	1.0000	1.0000
31	Phenol ethers	227	1	0.4	0.262	0.2310	1.0000	1.0000
32	Cinnamic acids and derivatives	300	1	0.4	0.346	0.2930	1.0000	1.0000
33	Coumarins and derivatives	341	1	0.4	0.394	0.3260	1.0000	1.0000
34	Diazines	342	1	0.4	0.395	0.3270	1.0000	1.0000
35	Prenol lipids	3830	3	1.2	4.42	0.8200	1.0000	1.0000
36	Flavonoids	1800	1	0.4	2.08	0.8760	1.0000	1.0000
37	Glycerophospholipids	40000	3	1.2	46.2	1.0000	1.0000	1.0000
