# Supplementary material for: Direct 3D Mass Spectrometry Imaging Analysis of Environmental Microorganisms
Source: Molecules. 2025 Mar 14;30(6):1317. doi: 10.3390/molecules30061317 (PMC11946574; doi:10.3390/molecules30061317)
Supplement: Supplementary file 1 [file molecules-30-01317-s001.zip › Table S9.pdf]

Table S9. LC-MS data supporting generated LARAPPI/CI-MSI 2D and LARAPPI/CI-MSI 3D ion images

Name	Molecular formula	Ion polarity	Formula of detected ions	RT [s]	*m/z*$_{meas.}$[a]	M$_{meas.}$[b]	Δ*m/z* [ppm]	ΔRT [s]	MS/MS score
2-Furoic acid	$C_5H_4O_3$	POS	$[M+NH_4]^+$	348.58	130.0496	112.0158	-1.919	15.70	-
Aminoadipic acid	$C_6H_{11}NO_4$	POS	$[M+H+CH_3CN]^+$	68.09	203.1026	161.0688	0.015	-	-
Asp-Leu	$C_{10}H_{18}N_2O_5$	POS	$[M+H]^+$	325.86	247.1286	246.1214	-0.894	-	976.8
Azelaic acid	$C_9H_{16}O_4$	POS	$[M+Na]^+$	506.48	211.0946	188.1054	2.549	10.34	
Citric acid	$C_6H_8O_7$	NEG	$[M-H]^-$	124.85	191.02	192.0273	1.145	2.57	-
Citrulline	$C_6H_{13}N_3O_3$	POS	$[M+NH_4-H_2O]^+$, $[M+H]^+$	64.59	175.1186	175.0953	-2.229	1.89	-
Deoxyguanosine	$C_{10}H_{13}N_5O_4$	NEG	$[M+HCOO]^-$	271.71	312.0954	267.0972	0.787	-2.19	
Diaminopimelic acid	$C_7H_{14}N_2O_4$	POS	$[M+H]^+$, $[M+K]^+$, $[M+Na]^+$	66.3	191.10284	190.0960	1.055	8.82	-
Elaidic acid	$C_{18}H_{34}O_2$	POS	$[M+Na]^+$	1022.47	305.245	282.2558	0.048	-	-
Fumaric acid	$C_4H_4O_4$	NEG	$[M-H]^-$	71.32	115.0038	116.0111	1.168	-	630.6
Indole-3-carboxylic acid	$C_9H_7NO_2$	NEG	[M+OH]-	496.08	178.0514	161.0481	2.591	9.78	
Kynurenic acid	$C_{10}H_7NO_3$	NEG	$[M+OH]^-$	495.37	178.0514	161.0482	2.643	9.07	
L-Lactic acid	$C_3H_6O_3$	POS	$[M+H+CH_3CN]^+$	73.95	132.0653	90.03145	-1.852	-8.01	-
L-Arginine	$C_6H_{14}N_4O_2$	POS	$[M+H]^+$, $[M+K]^+$	57.93	175.1187	174.1116	-1.623	1.53	
L-Aspartic acid	$C_4H_7NO_4$	POS	$[M+Na]^+$	64.43	156.0265	133.0373	-1.58	1.25	-
Leu-Pro	$C_{11}H_{20}N_2O_3$	POS	$[M+H]^+$	352.83	229.1544	228.1471	-1.655	-	995.5
L-Glutamic acid	$C_5H_9NO_4$	POS	[M+H]+, [M+H-H2O]+, [M+NH4-H2O]+	79.95	148.0602	147.0530	-1.671	-0.69	
L-Glutamine	$C_5H_{10}N_2O_3$	NEG	$[M-H]^-$	64.41	132.0305	133.0378	2.106	1.23	
Linoleic acid	$C_{18}H_{32}O_2$	NEG	$[M+HCOO]^-$	908.89	325.2386	280.2404	-0.368		
Linolenic acid	$C_{18}H_{30}O_2$	POS	$[M+H]^+$, $[M+H+CH_3CN]^+$, $[M+H-H_2O]^+$, $[M+Na]^+$	1031.21	279.2316	278.2235	-1.509	-	923.8
L-Proline	$C_5H_9NO_2$	POS	$[M+H]^+$	67.4	116.0704	115.0631	-2.115	0.26	-
L-Tryptophan	$C_{11}H_{12}N_2O_2$	NEG	$[M-H]^-$	361.54	203.0828	204.0900	0.815	13.96	-
Malic acid	$C_4H_6O_5$	NEG	$[M-H]^-$	78.82	133.0145	134.0217	1.526	0.64	

N-Acetylglycine	$C_4H_7NO_3$	NEG	$[M-H]^-$	139.31	116.0355	117.0427	1.127	-	1000
N-Acetylmannosamine	$C_8H_{15}NO_6$	NEG	$[M+Cl]^-$	67.66	256.0597	221.0903	1.523	0.04	-
N-Methylbenzamide	C_8H_9NO	POS	$[M+H]^+$	191.74	136.0753	135.0681	-2.633	-	372.5
Pantothenic acid	$C_9H_{17}NO_5$	NEG	$[M-H]^-$	619.14	223.098	224.1052	1.678		393.3
Pentadecanoic acid	$C_{15}H_{30}O_2$	POS	$[M+H-H_2O]^+$, $[M+H]^+$	941.78	225.22113	242.2242	-0.716	-	-
Pro-Asn	$C_9H_{15}N_3O_4$	POS	$[M+H]^+$	209.07	230.1134	229.1061	-0.734	-	809.0
Pro-Leu	$C_{11}H_{20}N_2O_3$	POS	$[M+H]^+$, $[M+Na]^+$	335.94	229.1544	228.1475	-1.267	-	997.9
Propionic acid	$C_3H_6O_2$	POS	$[M+H+CH_3CN]^+$	321.49	116.0704	74.0365	-2.216	9.31	
Pro-Pro	$C_{10}H_{16}N_2O_3$	POS	$[M+H]^+$	69.63	213.1232	212.1159	-1.155	-	984.1
Pro-Val	$C_{10}H_{18}N_2O_3$	POS	$[M+H]^+$	349.46	215.1388	214.1315	-0.755	-	973.6
PyroGlu-Ala	$C_8H_{12}N_2O_4$	POS	$[M+H]^+$	222.48	201.0869	200.0796	-0.459	-	650.7
Pyrrolidonecarboxylic acid	$C_5H_7NO_3$	POS	$[M+H]^+$	139.16	130.0496	129.0424	-1.123	-	-
Ribitol	$C_5H_{12}O_5$	POS	$[M+Na]^+$	65.85	175.0575	152.0682	-1.407	3.15	-
Sebacic acid	$C_{10}H_{18}O_4$	POS	$[M+Na+CH_3CN]^+$	528.81	266.1363	202.1205	0.296	-16.95	-
Sorbitol	$C_6H_{14}O_6$	NEG	$[M-H]^-$, $[M+HCOO]^-$	62.77	181.0720	182.0793	1.426	0.31	-
Succinic acid	$C_4H_6O_4$	POS	$[M+Na]^+$	135.41	141.0158	118.0266	0.395	-2.59	-
Trehalose	$C_{12}H_{22}O_{11}$	POS	$[M+Na]^+$	74.41	365.1052	342.1160	-0.696	0.01	-
Valeric acid	$C_5H_{10}O_2$	POS	$[M+H]^+$	488.28	103.0753	102.0680	-0.669	3.12	-
Xanthurenic acid	$C_{10}H_7NO_4$	POS	$[M+H]^+$	351.08	206.0446	205.0373	-1.105	8.60	-

[a]experimental *m/z* of monoisotopic signal; [b]experimental neutral monoisotopic mass
